# Multivariate genome-wide association study of depression, cognition, and memory phenotypes and validation analysis identify 12 cross-ethnic variants

**DOI:** 10.1038/s41398-022-02074-x

**Published:** 2022-07-30

**Authors:** Jing Sun, Weijing Wang, Ronghui Zhang, Haiping Duan, Xiaocao Tian, Chunsheng Xu, Xue Li, Dongfeng Zhang

**Affiliations:** 1 Department of Epidemiology and Health Statistics, The School of Public Health of Qingdao University, Qingdao, Shandong Province China; 2 Department of Big Data in Health Science School of Public Health, Center of Clinical Big Data and Analytics of The Second Affiliated Hospital, Zhejiang University School of Medicine, Hangzhou, China; 3 Qingdao Municipal Center for Disease Control and Prevention, No. 175 Shandong Road, Shibei District, Qingdao, Shandong Province China

**Keywords:** Genomics, Psychiatric disorders

## Abstract

To date, little is known about the pleiotropic genetic variants among depression, cognition, and memory. The current research aimed to identify the potential pleiotropic single nucleotide polymorphisms (SNPs), genes, and pathways of the three phenotypes by conducting a multivariate genome-wide association study and an additional pleiotropy analysis among Chinese individuals and further validate the top variants in the UK Biobank (UKB). In the discovery phase, the participants were 139 pairs of dizygotic twins from the Qingdao Twins Registry. The genome-wide efficient mixed-model analysis identified 164 SNPs reaching suggestive significance (*P* < 1 × 10^−5^). Among them, rs3967317 (*P* = 1.21 × 10^−8^) exceeded the genome-wide significance level (*P* < 5 × 10^−8^) and was also demonstrated to be associated with depression and memory in pleiotropy analysis, followed by rs9863698, rs3967316, and rs9261381 (*P* = 7.80 × 10^−8^−5.68 × 10^−7^), which were associated with all three phenotypes. After imputation, a total of 457 SNPs reached suggestive significance. The top SNP chr6:24597173 was located in the *KIAA0319* gene, which had biased expression in brain tissues. Genes and pathways related to metabolism, immunity, and neuronal systems demonstrated nominal significance (*P* < 0.05) in gene-based and pathway enrichment analyses. In the validation phase, 12 of the abovementioned SNPs reached the nominal significance level (*P* < 0.05) in the UKB. Among them, three SNPs were located in the *KIAA0319* gene, and four SNPs were identified as significant expression quantitative trait loci in brain tissues. These findings may provide evidence for pleiotropic variants among depression, cognition, and memory and clues for further exploring the shared genetic pathogenesis of depression with Alzheimer’s disease.

## Introduction

Depression and Alzheimer’s disease (AD) are two common mental disorders that pose a serious threat to the physical and mental health of the public, especially elderly persons [1, 2], resulting in a heavy burden of disease worldwide [3, 4]. An increasing number of studies support that depression and AD are comorbidities [5, 6]. Evidence has shown some common pathogenic mechanisms and pathological changes between depression and AD, such as chronic inflammation [7, 8], dysregulation of the hypothalamic-pituitary-adrenal axis [9], lower levels of norepinephrine and 5-hydroxytryptamine [10], and a smaller volume of the hippocampus [11], suggesting the possibility and biological rationality of the basis of comorbidity between depression and AD.

In addition to environmental factors, genetic factors play an important etiologic role in both depression and AD. According to a meta-analysis of twin studies, the heritability of depression ranges from 31% to 42% [12]. As the most commonly used endophenotypes of AD in genetic studies [13, 14], the heritability of cognition and memory ranges from 47% to 59% and 36% to 47%, respectively, based on a large meta-analysis of twin and family studies [15]. Moreover, some evidence has suggested a shared genetic basis among depression, cognition, and memory. Pertinently, Franz et al. found that shared genetic effects could explain 77% of the correlation of early cognitive function with midlife depression in American male twins [16]. Another study in male twins showed that the genetic correlation of executive function with the genetic effects shared by persons with depression and anxiety was −0.44 [17]. The Colorado Adoption Project’s study revealed that the genetic correlations of memory with several dimensions of cognition ranged from 0.59 to 0.69 [18]. Additionally, our previous study utilizing multivariate twin models found that, among Qingdao twins, the genetic correlations were −0.31 for depression and cognition, −0.28 for depression and memory, and 0.69 for memory and cognition, respectively [19].

However, to date, little is known about the shared genetic variants among depression, cognition, and memory. A depression genome-wide meta-analysis identified 269 genes associated with depression [20]. Interestingly, 70 of the 269 depression-related genes were also found in another genome-wide meta-analysis of cognitive function [21]. In addition, the *hNP* gene was associated with both depression [22] and memory [23]. The *APOE-4* allele gene was linked to lower cognitive ability, faster cognitive decline [24], and poorer memory [25]. Studies reported that *DISC1* gene polymorphisms were associated with depression [26, 27], cognition [27], and memory [27, 28]. These overlapping findings from univariate studies provided indirect clues about the potentially shared susceptibility genes for depression, cognition, and memory, but there still has been a lack of direct evidence for the shared genetic variants and genes among the three phenotypes. Multivariate genome-wide association studies (GWASs) can be performed to search for potential pleiotropic genetic variants affecting multiple phenotypes by jointly modeling all phenotypes simultaneously rather than focusing on the simple overlap of genetic variants among different studies. Moreover, multivariate GWASs have higher statistical power and more accurate parameter estimation than univariate GWASs, which is helpful for the discovery of pleiotropic and small effects of genes [29, 30]. Notably, twins are particularly valuable for genetic studies owing to their sharing of rearing and intrauterine environments, as well as genetic similarity and discrepancy [31]. The combination of twin-based design with GWAS is excellent in controlling population stratification and ‘passive gene-environment correlation (*r*GE)’ and can distinguish direct genetic effects from indirect genetic effects [32]. Additionally, owing to the severe European bias of GWASs, it is unknown how many genetic risk loci can be translated across ethnicities [33]. Human genomics research among under-represented populations and cross-ethnic studies are urgently needed, given that cross-ethnicity generalizability is vital for improving genetic risk prediction and the applicability of therapeutic targets, alleviating bias and unfairness against specific subpopulations [34].

Thus, we performed a multivariate GWAS among Qingdao twins in China to explore the potential pleiotropic single nucleotide polymorphisms (SNPs), genes, and pathways among depression, cognition, and memory. An additional pleiotropy analysis was also performed for interpreting the possibility of pleiotropy. Then, to determine if these findings in Chinese can be generalized to different ethnic groups (cross-ethnicity generalizability), we further validated the top variants in an independent UK Biobank (UKB) population to identify cross-ethnic associations.

## Materials and methods

### Study population

In the discovery phase, the participants were adult twins from the Qingdao Twins Registry, China, and the details have been described in previous literature [19]. Blood samples were collected from participants after they fasted overnight, and identification of zygosity was carried out by sex, blood type, and microsatellite DNA gene scanning and typing. Participants who were monozygotic twins, were lactating or pregnant, had serious diseases or lacked biological sample information were excluded. Finally, the current multivariate GWAS sample included 139 dizygotic twin pairs.

### Phenotypes

Depression was assessed using the 30-item Geriatric Depression Scale (GDS-30, Chinese version), which consists of 30 questions, with an overall score of 0-30 points, and a higher score indicated more severe depressive symptoms. The GDS-30 is especially suitable for the assessment of depression in middle-aged and elderly individuals and is also highly valid in the Chinese population [35, 36].

Cognition was measured using the Montreal Cognitive Assessment (MoCA, Chinese version) with high reliability and acceptance in Chinese adults [37, 38]. This assessment involved attention, naming, delayed recall, language, visuospatial/executive ability, orientation, and abstraction, with a total score of 30 points. To correct for the effect of education on cognitive performance, education-adjusted scores were used [39], where the scores of participants with ≤12 years of education were given one additional point, but with a total score of no more than 30. A lower cognition score indicated worse cognitive ability.

Memory was assessed by the backward and forward digit span tasks of the Wechsler Adult Intelligence (WAIS, Chinese version). The total score ranging from 0-17 was obtained by summing the scores of backward and forward digit span, and a lower score indicated worse memory. Digit span tasks have widely been used to reflect short-term memory, and the backward digit span also reflects working memory [40].

### Genotyping, quality control, and imputation

Infinium Omni2.5Exome-8v1.2 BeadChip from Illumina was used for genotyping in dizygotic twins. After quality control, 1,338,905 SNPs with calling rate >0.98, locus missing <0.05, the significance of Hardy-Weinberg equilibrium (HWE) significance >1 × 10^−4^, and minor allele frequency (MAF) > 0.05 were included in this multivariate GWAS.

On the basis of the linkage disequilibrium (LD) principle, IMPUTE2 software [41] was utilized to impute untyped SNPs with reference to the data collected during the third phase of the 1000 Genomes Project (ASIAN) [42]. After filtering by HWE > 1 × 10^−4^, MAF > 0.05, and R^2^ > 0.6, a total of 7,399,084 SNPs were finally used in the post-imputation multivariate GWAS.

### Multivariate GWAS

#### SNP-based analysis

The genome-wide efficient mixed-model association (GEMMA) [43] was used to evaluate the association of SNP genotypes with depression-cognition-memory phenotypic pairs after depression, cognition, and memory scores were transformed by rank transformation based on Blom’s formula [44] to normalize their skewed distributions, adjusting for sex, age, and the first five genetic principal components (PCs). The GEMMA fitted a multivariate linear mixed model (mvLMM) for testing marker associations with multiple phenotypes (depression, cognition, and memory) simultaneously while controlling for relatedness (here intra-pair correlation of twins) and population structure. The significance level was defined as a *P* value threshold of <5 × 10^−8^ (a conventional Bonferroni-corrected threshold) [45], and the suggestive level was defined as a *P* value threshold of <1 × 10^−5^ (a commonly utilized threshold in GWAS) [46]. Quantile-quantile (Q-Q) and Manhattan plots were used to visualize the results. Furthermore, enhancer enrichment analysis was performed by submitting the list of the top 100 SNPs (ranked by *P* values) associated with depression-cognition-memory to HaploReg v4.1 [47], and the cell-type enhancers with a *P* value of <0.05 were reported. All genomic coordinates were based on human genome Build 37 (NCBI GRCh37).

For interpreting the possibility of pleiotropy, we further performed a pleiotropy analysis by using the R package “pleio” [48] to test which phenotypes were associated with the potential pleiotropic genetic variants with suggestive significance in the current multivariate GWAS. Specifically, a new likelihood-ratio test with an extended sequential approach was used to test pleiotropy, which provides a testing framework to identify the number of phenotypes associated with a genetic variant, accounting for correlations among the phenotypes [48]. First, the sequential tests of pleiotropy started at the null hypothesis that all coefficients were equal to zero (test 0). If this test 0 was rejected, then test 1 was performed, which allowed one coefficient to be non-zero to test whether the remaining coefficients were equal to zero. If the test 1 was rejected, we then performed the test 2, which allowed two non-zero coefficients, considering all possible combinations of two non-zero coefficients and testing whether the remaining coefficients were equal to zero. Whenever a *P* value greater than 0.05 was derived, the sequential testing stopped. If the *P* value of test 2 remained <0.05, it implied that all three phenotypes were associated with this variant.

#### Gene-based analysis

VEGAS2 [49] was applied to carry out gene-based analysis by integrating the SNPs within a gene, and “1000 G East Asian Population” was used. A *P* value of <2.61 × 10^−6^ (0.05/19,152) was regarded as a significant threshold by Bonferroni correction due to the 19,152 genes tested, and the nominal significance level was defined as a *P* value of <0.05 [50].

#### Pathway enrichment analysis

PASCAL [51] was utilized to evaluate pathway scores. SNPs were mapped to genes, and then the joint score of all genes involved in a pathway was calculated. The chi-squared and empirical scores were utilized to assess pathway enrichment of high-scoring genes. The Reactome, KEGG, and BioCarta databases were used to obtain pathway information. An emp-*P* value of <4.64 × 10^−5^ (0.05/1,077) was regarded as a significant threshold by Bonferroni correction due to the 1,077 pathways tested, and the nominal significance level was defined as an emp-*P* value of <0.05.

### Validation analysis

To identify the cross-ethnicity generalizability and cross-ethnic associations, we validated the top variants in an independent UKB population, which is a population-based cohort of 488,377 individuals with genotypic data across the United Kingdom; more details of genotyping, quality control, and imputation have been described elsewhere [52]. The phenotypic and genotypic data utilized in the current study were obtained from the third version of UKB data under an approved data application (application number: 66354). Depression was assessed using the two-item Patient Health Questionnaire (PHQ-2), and a total score of three and more indicated possible depression [53]. Cognition was measured through 13 numerical and verbal reasoning questions reflecting reasoning ability, and correct scores ranged from 0–13. Memory was assessed by the digit span task, and the maximum digits remembered correctly ranged from 2–12. A total of 46,102 individuals participated in and completed the depression, cognition, and memory tests. Cases (*n* = 355) were defined as participants with depression scores ≥3 and cognition and memory performance scores lower than the 25th percentile of their score distributions. Controls (*n* = 1775) were selected by matching individuals’ age and sex (ratio = 1:5) with those of the participants (*n* = 30,470) with depression scores <3 and cognition and memory performance ≥25th percentile of their score distributions. Finally, 2130 individuals (355 cases and 1775 controls) with a median age (interquartile range) of 55 (15) years were included in the validated sample. The top SNPs were validated by logistic regression analysis of the additive effect model, adjusting for the first 10 genetic PCs. A total of 469 of 481 SNPs (the union number before and after imputation) with *P* values lower than 1 × 10^−5^ in the discovery set were typed in the UKB data set and selected for validation. Thus, a *P* value < 1.07 × 10^−4^ (0.05/469) was regarded as a significant threshold by Bonferroni correction, and the nominal significance level was defined as a *P* value <0.05. Statistical analyses were performed utilizing R version 4.1.0.

### Expression quantitative trait loci (eQTL) analysis

**Fig. 1:**
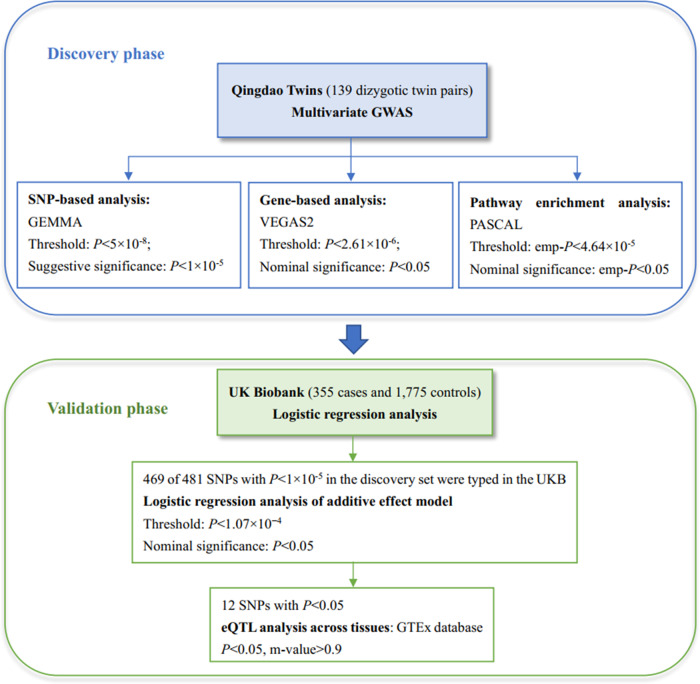
Flowchart of the overall study design and analysis steps.

For SNPs with nominal significance in the validation set, we further checked their functional consequences by eQTL analysis across tissues using data from the GTEx portal (version 8) [54]. A *P* value lower than 0.05 was regarded as significant in the single-tissue eQTL analysis. The posterior probability m-value that the eQTL effect existed in each tissue of a cross-tissue meta-analysis higher than 0.9 indicated that the tissue had an eQTL effect [55]. An outline of the overall study design and analysis steps is shown in Fig. 1.

## Results

### Basic characteristics

There were 139 pairs of dizygotic twins in the final discovery sample. The median (interquartile range) age was 49 (11) years, and the median scores (interquartile ranges) for depression, cognition, and memory for participants were 7 (7), 22 (5), and 12 (3) points, respectively (Supplementary Table 1).

### Multivariate GWAS

#### SNP-based analysis

**Fig. 2:**
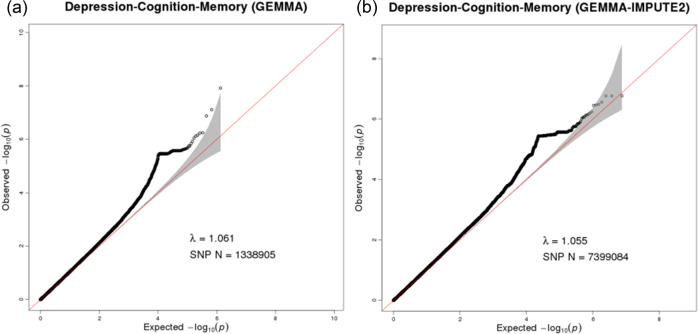
Quantile-quantile plot for multivariate genome-wide association study of depression-cognition-memory. **a** The quantile-quantile plot based on data before imputation. **b** The quantile-quantile plot based on data after imputation. The horizontal axis represents the expected −log_10_ (*P*), while the vertical axis represents the observed −log_10_ (*P*). The red line represents the expectation of the null hypothesis of no association, and the gray shaded area represents 95% confidence intervals of the null hypothesis. The black dots represent the observed data, and λ indicates genomic inflation.

**Fig. 3:**
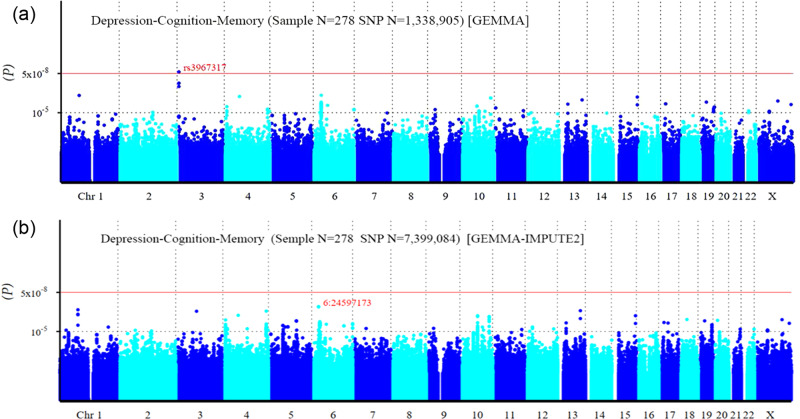
Manhattan plot for multivariate genome-wide association study of depression-cognition-memory. **a** The Manhattan plot based on data before imputation. **b** The Manhattan plot based on data after imputation. The horizontal axis represents autosomes and the X chromosome, while the vertical axis represents the *P* values of SNPs. The red line represents the genome-wide significance threshold (5 × 10^−8^), and the lower horizontal dashed line represents the suggestive significance level (1 × 10^−5^).

In the SNP-based study, the Q-Q plot (Fig. 2a) suggested no evidence of population stratification. The Manhattan plot (Fig. 3a) demonstrated that a total of 164 SNPs reached the level of suggestive significance (*P* < 1 × 10^−5^) (Supplementary Table 2); among them, rs3967317 (*P* = 1.21 × 10^−8^) on the *CNTN4* gene on chromosome 3 exceeded the genome-wide significance level (*P* < 5 × 10^−8^). In addition, rs9863698 (*P* = 7.80 × 10^−8^) and rs3967316 (*P* = 1.33 × 10^−7^) on the *CNTN4* gene, rs9261381 (*P* = 5.68 × 10^−7^) on the *TRIM31* gene, rs11577464 (*P* = 5.82 × 10^−7^) on the *LINC02567* gene, and rs73198369 (*P* = 7.00 × 10^−7^) on the *RNU6-1325P* gene reached suggestive significance. The top 20 SNPs ranked by *P* values are shown in Table 1. The additional pleiotropy analysis identified that 144 of the 164 SNPs (87.8%) were associated with two or three phenotypes (*P* < 0.05), with rs3967317 associated with depression and memory, rs9863698, rs3967316, and rs9261381 associated with all three phenotypes (Supplementary Table 2).

**Table 1:** The top 20 SNPs from multivariate GWAS of depression-cognition-memory.

SNP	Chr	Band	BP	*P* value	Gene or nearest gene	Official full name
**rs3967317**	3	3p26.3-p26.2	3058707	1.21E-08	*CNTN4*	contactin 4
**rs9863698**	3	3p26.3-p26.2	3059373	7.80E-08	*CNTN4*	contactin 4
**rs3967316**	3	3p26.3-p26.2	3062578	1.33E-07	*CNTN4*	contactin 4
**rs9261381**	6	6p22.1	30060002	5.68E-07	*TRIM31*	tripartite motif containing 31
rs11577464	1	1p31.1	77259107	5.82E-07	*LINC02567*	long intergenic non-protein coding RNA 2567
rs73198369	4	4q13.1	60865968	7.00E-07	*RNU6-1325P*	RNA, U6 small nuclear 1325, pseudogene
**rs8036389**	15	15q26.2	97492430	7.59E-07	*RN7SKP181*	RN7SK pseudogene 181
rs58350164	10	10q26.11	120568419	9.01E-07	*CACUL1*	CDK2 associated cullin domain 1
rs9589470	13	13q31.3	92917856	1.19E-06	*GPC5*	glypican 5
rs7056938	23	Xq21.31	87688360	1.45E-06	*CPXCR1*	CPX chromosome region candidate 1
rs114941840	19	19p12	22443558	1.76E-06	*LOC107985324*	uncharacterized LOC107985324
rs9260936	6	6p22.1	29958502	1.77E-06	*DDX39BP2*	DEAD-box helicase 39B pseudogene 2
rs9260918	6	6p22.1	29948751	2.10E-06	*MICD*	MHC class I polypeptide-related sequence D (pseudogene)
rs9260931	6	6p22.1	29957508	2.15E-06	*DDX39BP2*	DEAD-box helicase 39B pseudogene 2
rs9260672	6	6p22.1	29924996	2.23E-06	*HLA-W*	major histocompatibility complex, class I, W (pseudogene)
rs9260733	6	6p22.1	29932433	2.31E-06	*HLA-W*	major histocompatibility complex, class I, W (pseudogene)
rs4985694	17	17p11.2	16861332	2.36E-06	*TNFRSF13B*	TNF receptor superfamily member 13B
rs72835988	17	17p11.2	16868676	2.36E-06	*TNFRSF13B*	TNF receptor superfamily member 13B
rs3823382	6	6p22.1	29945210	2.45E-06	*HCG9*	HLA complex group 9
rs1331848	13	13q12.3	31508724	2.46E-06	*TEX26*; *TEX26-AS1*	testis expressed 26; TEX26 antisense RNA 1

*SNP* nucleotide polymorphism, *Chr* chromosome, *BP* base pair. The content discussed in detail or SNPs with nominal significance in UK Biobank were in bold.

Enhancer enrichment analysis found that the top 100 depression-cognition-memory-related genetic variants were significantly enriched in six tissues and cells, including pancreatic islets, stomach mucosa, fetal intestine large, primary natural killer cells, and T regulatory cells from peripheral blood, and liver (*P* < 0.05) (Supplementary Table 3).

After using the data of the third phase of the 1000 Genomes Project as a reference to impute untyped SNPs, there was still no evidence of population stratification (Fig. 2b), and the amounts of SNPs with suggestive significance increased, with a total of 457 SNPs reaching the level of suggestive significance (Fig. 3b, Supplementary Table 4). The first three top SNPs, chr6:24597173, rs12210323, and rs12213116 (*P* = 1.71 × 10^−7^−1.72 × 10^−7^), were located in the *KIAA0319* gene, which had biased expression in multiple brain tissues (Supplementary Fig 1), followed by rs61783213 (*P* = 2.77 × 10^−7^) in the *LINC02567* gene. The top 20 SNPs after imputation are shown in Supplementary Table 5. A total of 377 of the 457 SNPs (82.5%) were associated with two or three phenotypes (*P* < 0.05) (Supplementary Table 4).

#### Gene-based analysis

In the gene-based study, no statistically significant gene was found (*P* < 2.61 × 10^−6^), but 1107 genes reached the nominal significance level (*P* < 0.05). Most of these genes are known to be involved in metabolism, immunity, and neuronal systems, and the top 20 genes are shown in Table 2.

**Table 2:** The top 20 genes associated with depression-cognition-memory from gene-based analysis.

Gene	Chr	SNP (N)	Start	Stop	*P* value	Top-SNP	Top-SNP *P* value
*TMEM257*	23	2	144908927	144911370	1.00E-05	rs5919909	2.59E-06
*TEX26*	13	21	31506833	31549153	2.10E-05	rs1331848	2.46E-06
***MEDAG***	13	14	31480311	31499709	4.80E-05	rs10742	5.35E-05
*LSM10*	1	5	36859030	36863493	6.80E-05	rs4653185	2.07E-05
*ZMAT4*	8	200	40388110	40755343	9.10E-05	rs28603251	3.45E-05
*KIAA0232*	4	31	6784458	6885899	9.50E-05	rs3893185	3.69E-06
*VDAC3*	8	5	42249278	42263455	1.47E-04	rs59791694	2.17E-04
*CTD-2297D10.2*	5	17	5132197	5140167	2.63E-04	rs4702210	1.53E-05
*OR6K6*	1	2	158724605	158725637	3.21E-04	rs16841001	6.69E-05
*SLC26A7*	8	62	92221721	92410382	3.49E-04	rs10101012	8.61E-04
*TEX26-AS1*	13	37	31456971	31506745	3.84E-04	rs10742	5.35E-05
***C3***	19	38	6677845	6720662	4.03E-04	rs2250656	7.69E-04
***TET1***	10	56	70320116	70454239	4.39E-04	rs7912984	7.30E-06
*LOC102724316*	10	19	29698462	29776785	4.47E-04	rs6481596	3.22E-05
*OR6N1*	1	5	158735533	158736472	5.41E-04	rs857826	3.86E-04
*IQCJ*	3	74	158787040	158984096	5.76E-04	rs10470473	1.91E-04
*TBC1D16*	17	113	77906141	78009657	5.84E-04	rs12602951	3.47E-04
*WDPCP*	2	86	63348534	63815867	6.02E-04	rs6545988	7.39E-05
*USP12-AS1*	13	14	27699669	27743272	6.08E-04	rs9512556	8.12E-04
*CATSPERG*	19	17	38826442	38861589	6.66E-04	rs2270095	2.03E-04

*Chr* chromosome, *SNP* nucleotide polymorphism. The content discussed in detail were in bold.

#### Pathway enrichment analysis

In the pathway-based analysis, no statistically significant pathway was found (*P* < 4.64 × 10^−5^), but 587 pathways were found to be nominally associated with depression-cognition-memory (emp-*P* < 0.05), and most of these pathways were involved in the metabolism of amino acids, lipids and RNA, the immune system, and the neuronal system. The top 20 pathways are shown in Table 3.

**Table 3:** The top 20 pathways associated with depression-cognition-memory from pathway enrichment analysis.

Pathway	chisq-*P*	emp-*P*	−log (chisq-*P*)	−log (emp-*P*)
REACTOME_METABOLISM_OF_AMINO_ACIDS_AND_DERIVATIVES	6.50E-04	2.42E-04	3.19	3.62
REACTOME_SPHINGOLIPID_DE_NOVO_BIOSYNTHESIS	1.08E-03	2.65E-04	2.97	3.58
REACTOME_SLBP_DEPENDENT_PROCESSING_OF_REPLICATION_DEPENDENT_HISTONE_PRE_MRNAS	6.63E-04	3.95E-04	3.18	3.40
REACTOME_PROCESSING_OF_CAPPED_INTRONLESS_PRE_MRNA	6.63E-04	4.18E-04	3.18	3.38
KEGG_SPHINGOLIPID_METABOLISM	1.22E-03	5.30E-04	2.91	3.28
REACTOME_N_GLYCAN_ANTENNAE_ELONGATION_IN_THE_MEDIAL_TRANS_GOLGI	1.84E-03	7.70E-04	2.74	3.11
REACTOME_N_GLYCAN_ANTENNAE_ELONGATION	1.84E-03	1.03E-03	2.74	2.99
REACTOME_IMMUNOREGULATORY_INTERACTIONS_BETWEEN_A_LYMPHOID_AND_A_NON_LYMPHOID_CELL	1.46E-03	1.24E-03	2.83	2.91
REACTOME_TRANSCRIPTIONAL_REGULATION_OF_WHITE_ADIPOCYTE_DIFFERENTIATION	3.12E-03	1.41E-03	2.51	2.85
REACTOME_NONSENSE_MEDIATED_DECAY_ENHANCED_BY_THE_EXON_JUNCTION_COMPLEX	2.42E-03	1.43E-03	2.62	2.84
REACTOME_TRANSPORT_TO_THE_GOLGI_AND_SUBSEQUENT_MODIFICATION	1.93E-03	1.47E-03	2.71	2.83
REACTOME_SIGNALING_BY_FGFR1_FUSION_MUTANTS	1.55E-03	1.66E-03	2.81	2.78
REACTOME_SIGNAL_REGULATORY_PROTEIN_SIRP_FAMILY_INTERACTIONS	1.89E-03	1.87E-03	2.72	2.73
REACTOME_OTHER_SEMAPHORIN_INTERACTIONS	1.89E-03	1.91E-03	2.72	2.72
REACTOME_DNA_REPAIR	5.00E-03	2.13E-03	2.30	2.67
REACTOME_TRANSFERRIN_ENDOCYTOSIS_AND_RECYCLING	3.30E-03	3.01E-03	2.48	2.52
BIOCARTA_NKCELLS_PATHWAY	4.69E-03	3.04E-03	2.33	2.52
REACTOME_SIGNALING_BY_FGFR3_MUTANTS	3.78E-03	3.06E-03	2.42	2.51
REACTOME_APOPTOTIC_EXECUTION_PHASE	5.75E-03	3.10E-03	2.24	2.51
REACTOME_CELL_JUNCTION_ORGANIZATION	6.99E-03	3.15E-03	2.16	2.50

#### Validation analysis

A total of 469 SNPs with *P* values lower than 1 × 10^−5^ in the discovery set had genotype data in the UKB validation set and were selected for validation. Although no SNP passed the Bonferroni correction level, 12 SNPs reached the nominal significance level (*P* < 0.05), three of them (rs13209442, rs13208577, and rs12213116) were located in the *KIAA0319* gene, and one (rs9261134) was located in the *ZNRD1ASP* gene (Supplementary Table 6).

#### eQTL analysis

The eQTL analysis across tissues found that four (rs2539731, rs17337582, rs62358383, and rs9261134) of 12 SNPs with nominal significance in the validation set were significant eQTLs in multiple tissues, specifically in brain tissues (Supplementary Figs 2–5). Among these SNPs, rs2539731 (Supplementary Fig 2, brain-substantia nigra: *P* = 1.10 × 10^−3^, m-value = 0.965; brain-nucleus accumbens: *P* = 3.60 × 10^−5^, m-value = 0.991), rs17337582 (Supplementary Fig 3, brain-substantia nigra: *P* = 1.20 × 10^−3^, m-value = 0.951; brain-nucleus accumbens: *P* = 4.10 × 10^−5^, m-value=0.996), and rs62358383 (Supplementary Fig 4, brain-substantia nigra: *P* = 1.90 × 10^−3^, m-value = 0.927; brain-nucleus accumbens: *P* = 5.70 × 10^−5^, m-value = 0.987) were significantly associated with the expression of *MAP3K1* gene in brain tissues. Rs9261134 located at the *ZNRD1ASP* gene was significantly associated with the expression of 15 genes (Supplementary Table 6) in multiple brain tissues (brain-nucleus accumbens, brain-frontal cortex, brain-caudate, brain-putamen, brain-hypothalamus, brain-amygdala, brain-cortex, and brain-anterior cingulate cortex) (*P* < 0.5, m-value > 0.9), and *ZNRD1ASP* expression with rs9261134 across tissues is shown in Supplementary Fig 5.

## Discussion

The current study performed the first multivariate GWAS of depression-cognition-memory and found some pleiotropic SNPs, genes, and pathways among depression, cognition, and memory in Qingdao twins. Moreover, multiple variants were replicated in another independent UKB population.

Although few previous achievements have been made involving pleiotropic variants of depression, cognition, and memory across ancestries, some recent literature has focused on both depression and cognitive function or depression related variants across ancestry groups. Thalamuthu et al. performed a genome-wide interaction analysis of major depressive disorder (MDD) with cognitive function among European cohorts. The study revealed that MDD status had a moderating effect on the associations of variants with cognitive function, with some SNPs associated with cognitive domains in the context of MDD [56]. Another study conducted pleiotropy analyses, utilizing MDD and late-onset AD GWAS data based on European ancestry, thereby indicating that the genetic risks associated with AD might influence MDD risk [57]. Cross-ethnic studies, similar to our findings, demonstrated a small shared polygenic basis for depression in European and East Asian populations. Bigdeli et al. found a weak overlap of SNP effects between East Asian and European ancestries by combining MDD GWAS summary statistics of Chinese and European participants [58]. One significant SNP (rs10912903) was replicated in the current multivariate GWAS, with a *P* value of 7.15 × 10^−3^. Another study also showed that only 11% of depression risk loci previously identified in the European population reached nominal significance in the East Asian population [59].

In the SNP-based analysis, the strongest association signal was rs3967317 located in the *CNTN4* gene on chromosome 3, which exceeded the genome-wide significance level. The following were rs9863698 and rs3967316, which were also located in the *CNTN4* gene. The *CNTN4*-encoded protein belongs to the contactin family and is involved in neuronal network development and plasticity. Pertinently, studies have found that *CNTN4* is associated with mental retardation [60] and affects intelligence [61]. Rs9261381 was located in the *TRIM31* gene on chromosome 6. *TRIM31* encodes a protein that functions as an E3 ubiquitin-protein ligase and can regulate cell growth. Studies have shown that the *TRIM31* gene was associated with intelligence only in the background of a psychiatric disorder [62]. Furthermore, the top 100 depression-cognition-memory related genetic variants were significantly enriched in pancreatic islets, stomach mucosa, fetal intestine large, primary natural killer cells and T regulatory cells from peripheral blood, and the liver. Ample evidence has shown that these tissues and cells are closely related to depression, cognition, and memory [63–70], which further supports our findings.

After imputing untyped SNPs, more SNPs with suggestive significance were identified. The SNP chr6:24597173 located in the *KLAA0319* gene showed the strongest association, although this might be mainly driven by the association of the SNP with cognition. However, the *KLAA0319* gene demonstrated a biased expression in multiple brain tissues, and three of 12 SNPs with nominal significance in the validation phase among the UKB population whose cases were abnormal on all three phenotypes and controls were normal were located in the *KIAA0319* gene, indicating a potential cross-ethnic association of *KIAA0319* with depression-cognition-memory. The protein encoded by *KIAA0319* can regulate cell adhesion and neuronal migration processes to influence the growth of the cerebral cortex, and *KIAA0319* has been found to be associated with category fluency, recall process, and verbal learning [71]. In addition, four of the other nine SNPs were significant eQTLs in brain tissues that control mood, cognition, and memory; moreover, three SNPs were significantly associated with the expression level of the *MAP3K1* gene in the brain-substantia nigra and brain-nucleus accumbens. Pertinently, the protein encoded by *MAP3K1* is part of the nuclear factor kappa beta (NF-κB) pathway [72]. NF-κB has been found to be involved in the pathophysiology of depression [73] and is closely related to the pathogenesis of AD [74].

Most genes with nominal significance levels in the gene-based analysis are known to be involved in metabolism, immunity, and neuronal systems. The potential mechanisms of several interesting genes other than those mentioned above were as follows: (1) *MEDAG* is an adipogenic gene that can promote the formation of adipocytes [75]. The lipid metabolism process involves the pathogenesis of depression and AD [76, 77]; (2) the C3a peptide encoded by *C3* can modulate the inflammation process which is closely related to depression and AD [7, 8]; and (3) the protein encoded by *TET1* influences gene activation and the process of DNA methylation. Studies have shown that the expression level of *TET1* is significantly increased in psychotic participants [78], and *TET1* variation is associated with late-onset AD [79]; (4) *FGF1* is related to the survival of neurons and is involved in various biological processes. Furthermore, *FGF1* has been reported to be associated with AD in Han Chinese individuals [80].

In the pathway enrichment analysis, most pathways that reached nominal significance were related to metabolism, immune, and neuronal system, and several important pathways were revealed as follows: (1) metabolism of amino acids and derivatives, various metabolites of this pathway including glutamate and glycine are involved in the pathophysiology of depression and AD [81, 82]; (2) sphingolipid de novo biosynthesis and (3) sphingolipid metabolism pathways, both of which are involved in the metabolism process of sphingolipid. The intermediate product is ceramide, which is closely related to the pathological mechanisms of depression and AD and used as a therapeutic target [83]. For (4) N-glycan antennae elongation in the medial/trans-Golgi and (5) N-glycan antennae elongation, there are significant differences between depression patients and controls in the serum N-glycan structure levels [84], and N-glycans can influence the development and progression of AD by regulating the key glycoproteins [85]. (6) Immunoregulatory interactions between a lymphoid and a non-lymphoid cell, which is an important pathway in the immune system; relevantly, the immune system is regarded as a major factor in both depression and AD [86, 87]. A previous study also found immune-related pathways in the shared genetic etiology of depression and AD [57]. (7) Other semaphorin interactions pathway containing four types of plexins and eight classes of semaphorins is involved in axon guidance and the development of the nervous system [88]. Semaphorins have been related to major depression risk [89], and plexin-A4 can mediate Aβ-induced tau pathology in the pathogenesis of AD [90]. However, some pathways, such as cortisol/stress responses and cholinergic and serotonergic function [10], have been clearly linked to both depression and AD but were not identified as top pathways in the current study with higher *P* values (stress pathway: emp-*P* = 2.60 × 10^−2^, neurotransmitter release cycle: emp-*P* = 2.16 × 10^−2^, acetylcholine neurotransmitter release cycle pathway: emp-*P* = 2.19 × 10^−2^, etc.). Except for the limitation of the small sample size, another possible reason was that although several physiological processes were thought to be the common mechanisms of both depression and AD, they might be mainly caused by different genes in depression and AD, rather than pleiotropic genes, and further research is required in the future.

The current multivariate GWAS had several strengths. First, this is the first study to identify potential pleiotropic SNPs, genes, and pathways among depression, cognition, and memory phenotypes in Chinese individuals, which may not only provide insight into a common genetic basis of these phenotypes but also make a little contribution in shifting the Eurocentric bias of GWASs. Second, this current GWAS was performed in twin samples. The twin-based GWAS design has been demonstrated excellence in controlling population stratification and passive *r*GE and can identify direct genetic effects [32], which reduced the concerns about false-positive errors and the confusion of indirect genetic effects. Third, validation analysis was performed in another independent UKB population, which allowed the identification of cross-ethnic generalizability. However, several study limitations should also be considered. First, the sample size of the current study was relatively small owing to the difficulty in twin pair recruitment, even though the algorithm of multivariate GWAS has the natural advantage of power [91], which may restrict the discovery of more significantly associated SNPs, genes, and pathways. In fact, no gene and pathway reached the significance threshold of Bonferroni correction, which might be partly due to the small sample size, and further research in a large East Asian population is needed. Notably, cognitive decline in depressed individuals could be secondary to depression itself, such as psychomotor slowing and withdrawal from engagement in activities that were conducive to cognition. Similarly, cognitive dysfunction might lead to stress and psychological burdens that give rise to depressive symptoms. In these cases, the genetics affecting cognition and memory might largely be separate from those that served as other risk factors for depression, which might also explain the lack of extensive genetic overlap among the three phenotypes in the current study. Second, the assessment methods of depression, cognition, and memory phenotypes were not perfectly consistent between the discovery set and validation set; for example, depression was assessed using the GDS-30 in the current multivariate GWAS and the PHQ-2 in UKB validation analysis. Although both the GDS-30 and PHQ-2 are reliable and valid and have been widely used as screening measures for depression, there may still be phenotypic heterogeneity to some degree due to the not identical questionnaire items. The phenotypic heterogeneity and ethnic difference in the MAF and LD structure might partly account for the fewer significant findings revealed in the validation analysis. Nevertheless, we still found a potential cross-ethnic association of *KIAA0319*. However, the strongest SNP (rs3967317), which demonstrated genome-wide significance (*P* < 5 × 10^−8^) and still showed suggestive significance after imputation (*P* < 1 × 10^−5^) in the discovery set, was not replicated in the UKB validation sample, but it may still be worth being further validated in the Asian population as a promising candidate variant.

In conclusion, this multivariate GWAS identified some pleiotropic SNPs, genes, and pathways among depression, cognition, and memory, which provided evidence for a common genetic basis of the three phenotypes and clues for further exploring the shared genetic pathogenesis of depression with AD, and it might be helpful in the search for new therapeutic targets for both diseases.

## Supplementary information

Supplementary Table 1

Supplementary Table 2

Supplementary Table 3

Supplementary Table 4

Supplementary Table 5

Supplementary Table 6

Supplementary figure and table legends

Supplementary Figure 1

Supplementary Figure 2

Supplementary Figure 3

Supplementary Figure 4

Supplementary Figure 5

## Data Availability

The dataset used in the present study is available from the corresponding author upon reasonable request.
