# Supplementary material for: Multivariate genome-wide association study of depression, cognition, and memory phenotypes and validation analysis identify 12 cross-ethnic variants
Source: Transl Psychiatry. 2022 Jul 30;12:304. doi: 10.1038/s41398-022-02074-x (PMC9338946; doi:10.1038/s41398-022-02074-x)
Supplement: Supplementary file 1 — Supplementary Table 1 [file 41398_2022_2074_MOESM1_ESM.doc]

**Supplementary Table 1** Basic characteristics of participants in the discovery phase

| Variable | Female | | Male | | All | |
| --- | --- | --- | --- | --- | --- | --- |
|  | N | M (Q) | N | M (Q) | N | M (Q) |
| Age (years) | 137 | 49 (11) | 141 | 50 (11) | 278 | 49 (11) |
| Depression score | 137 | 6 (6) | 141 | 7 (8) | 278 | 7 (7) |
| Cognition score | 137 | 23 (6) | 141 | 21 (6) | 278 | 22 (5) |
| Memory score | 137 | 12 (3) | 141 | 12 (3) | 278 | 12 (3) |

M (Q): median (interquartile range).
