# Supplementary material for: Multivariate genome-wide association study of depression, cognition, and memory phenotypes and validation analysis identify 12 cross-ethnic variants
Source: Transl Psychiatry. 2022 Jul 30;12:304. doi: 10.1038/s41398-022-02074-x (PMC9338946; doi:10.1038/s41398-022-02074-x)
Supplement: Supplementary file 2 — Supplementary Table 2 [file 41398_2022_2074_MOESM2_ESM.doc]

**Supplementary Table 2** The results of pleiotropy analysis for multivariate GWAS of depression-cognition-memory identified SNPs (*P*<1×10^-5^)

| SNP | Chr | BP | *P* value ^a^ | Trait of nonzero *β* ^b^ | *P* for test 0 ^c^ | *P* for test 1 ^d^ | Trait of nonzero *β* for test 1 ^d^ | *P* for test 2 ^e^ | Trait of nonzero *β* for test 2 ^e^ | Associated phenotype |
| --- | --- | --- | --- | --- | --- | --- | --- | --- | --- | --- |
| rs3967317 | 3 | 3058707 | 1.21E-08 | D; M | 3.58E-09 | 9.86E-03 | M | 9.83E-02 | D; M | D; M |
| rs9863698 | 3 | 3059373 | 7.80E-08 | D; C; M | 2.12E-08 | 2.98E-03 | M | 3.10E-02 | D; M | D; C; M |
| rs3967316 | 3 | 3062578 | 1.33E-07 | D; C; M | 4.79E-08 | 2.65E-03 | M | 2.86E-02 | C; M | D; C; M |
| rs9261381 | 6 | 30060002 | 5.68E-07 | D; C; M | 1.13E-07 | 3.90E-04 | D | 4.53E-02 | D; C | D; C; M |
| rs11577464 | 1 | 77259107 | 5.82E-07 | C; M | 2.52E-09 | 2.25E-05 | C | 9.32E-01 | C; M | C; M |
| rs73198369 | 4 | 60865968 | 7.00E-07 | M | 7.09E-07 | 2.88E-01 | M | - | - | M |
| rs8036389 | 15 | 97492430 | 7.59E-07 | D | 2.11E-04 | 1.44E-01 | D | - | - | D |
| rs58350164 | 10 | 120568419 | 9.01E-07 | D; C | 1.40E-06 | 5.25E-04 | D | 5.16E-01 | D; C | D; C |
| rs9589470 | 13 | 92917856 | 1.19E-06 | D; C; M | 7.07E-07 | 1.64E-03 | D | 2.71E-02 | D; C | D; C; M |
| rs7056938 | 23 | 87688360 | 1.45E-06 | C | 8.03E-08 | 7.37E-01 | C | - | - | C |
| rs114941840 | 19 | 22443558 | 1.76E-06 | C; M | 1.71E-07 | 1.33E-04 | M | 2.66E-01 | C; M | C; M |
| rs9260936 | 6 | 29958502 | 1.77E-06 | D; C | 3.50E-07 | 1.34E-03 | D | 1.06E-01 | D; C | D; C |
| rs9260918 | 6 | 29948751 | 2.10E-06 | D; C | 6.64E-07 | 2.53E-03 | D | 7.99E-02 | D; C | D; C |
| rs9260931 | 6 | 29957508 | 2.15E-06 | D; C | 3.50E-07 | 3.06E-03 | D | 2.22E-01 | D; C | D; C |
| rs9260672 | 6 | 29924996 | 2.23E-06 | D; C | 3.69E-07 | 3.21E-03 | D | 2.21E-01 | D; C | D; C |
| rs9260733 | 6 | 29932433 | 2.31E-06 | D; C | 5.93E-07 | 3.62E-03 | D | 2.25E-01 | D; C | D; C |
| rs4985694 | 17 | 16861332 | 2.36E-06 | C | 5.60E-07 | 1.92E-01 | C | - | - | C |
| rs72835988 | 17 | 16868676 | 2.36E-06 | C | 5.60E-07 | 1.92E-01 | C | - | - | C |
| rs3823382 | 6 | 29945210 | 2.45E-06 | D; C | 5.66E-07 | 3.77E-03 | D | 2.10E-01 | D; C | D; C |
| rs1331848 | 13 | 31508724 | 2.46E-06 | D; C; M | 1.00E-08 | 1.01E-05 | C | 4.43E-03 | C; M | D; C; M |
| rs5919909 | 23 | 144910142 | 2.59E-06 | D; C | 2.66E-06 | 1.66E-02 | D | 1.76E-01 | D; C | D; C |
| rs9260916 | 6 | 29948708 | 2.63E-06 | D; C | 6.57E-07 | 4.07E-03 | D | 2.25E-01 | D; C | D; C |
| rs2232239 | 6 | 29942866 | 2.65E-06 | D; C | 5.90E-07 | 3.79E-03 | D | 2.17E-01 | D; C | D; C |
| rs3823384 | 6 | 29945368 | 2.65E-06 | D; C | 5.90E-07 | 3.79E-03 | D | 2.17E-01 | D; C | D; C |
| rs3901554 | 6 | 29955025 | 2.65E-06 | D; C | 5.90E-07 | 3.79E-03 | D | 2.17E-01 | D; C | D; C |
| rs9260768 | 6 | 29937304 | 2.65E-06 | D; C | 5.90E-07 | 3.79E-03 | D | 2.17E-01 | D; C | D; C |
| rs9260830 | 6 | 29942979 | 2.65E-06 | D; C | 5.90E-07 | 3.79E-03 | D | 2.17E-01 | D; C | D; C |
| rs9260834 | 6 | 29943475 | 2.65E-06 | D; C | 5.90E-07 | 3.79E-03 | D | 2.17E-01 | D; C | D; C |
| rs9260860 | 6 | 29945508 | 2.65E-06 | D; C | 5.90E-07 | 3.79E-03 | D | 2.17E-01 | D; C | D; C |
| rs9260861 | 6 | 29945594 | 2.65E-06 | D; C | 5.90E-07 | 3.79E-03 | D | 2.17E-01 | D; C | D; C |
| rs9260862 | 6 | 29945741 | 2.65E-06 | D; C | 5.90E-07 | 3.79E-03 | D | 2.17E-01 | D; C | D; C |
| rs9260863 | 6 | 29945771 | 2.65E-06 | D; C | 5.90E-07 | 3.79E-03 | D | 2.17E-01 | D; C | D; C |
| rs9260864 | 6 | 29945841 | 2.65E-06 | D; C | 5.90E-07 | 3.79E-03 | D | 2.17E-01 | D; C | D; C |
| rs9260870 | 6 | 29946503 | 2.65E-06 | D; C | 5.90E-07 | 3.79E-03 | D | 2.17E-01 | D; C | D; C |
| rs9260873 | 6 | 29946656 | 2.65E-06 | D; C | 5.90E-07 | 3.79E-03 | D | 2.17E-01 | D; C | D; C |
| rs9260914 | 6 | 29948401 | 2.65E-06 | D; C | 5.90E-07 | 3.79E-03 | D | 2.17E-01 | D; C | D; C |
| rs9260915 | 6 | 29948557 | 2.65E-06 | D; C | 5.90E-07 | 3.79E-03 | D | 2.17E-01 | D; C | D; C |
| rs9260919 | 6 | 29948884 | 2.65E-06 | D; C | 5.90E-07 | 3.79E-03 | D | 2.17E-01 | D; C | D; C |
| rs9260923 | 6 | 29949889 | 2.65E-06 | D; C | 5.90E-07 | 3.79E-03 | D | 2.17E-01 | D; C | D; C |
| rs9260930 | 6 | 29956953 | 2.65E-06 | D; C | 5.90E-07 | 3.79E-03 | D | 2.17E-01 | D; C | D; C |
| rs9260933 | 6 | 29957866 | 2.65E-06 | D; C | 5.90E-07 | 3.79E-03 | D | 2.17E-01 | D; C | D; C |
| rs9260934 | 6 | 29957982 | 2.65E-06 | D; C | 5.90E-07 | 3.79E-03 | D | 2.17E-01 | D; C | D; C |
| rs2041433 | 15 | 97493539 | 2.67E-06 | D | 9.73E-05 | 3.14E-01 | D | - | - | D |
| rs9260989 | 6 | 29962517 | 2.80E-06 | D; C | 1.39E-06 | 9.08E-04 | D | 5.13E-02 | D; C | D; C |
| rs6924054 | 6 | 29967618 | 2.87E-06 | D; C | 1.47E-06 | 9.40E-04 | D | 5.35E-02 | D; C | D; C |
| rs75490575 | 6 | 29800795 | 2.87E-06 | D; C | 1.47E-06 | 9.40E-04 | D | 5.35E-02 | D; C | D; C |
| rs9261038 | 6 | 29966513 | 2.87E-06 | D; C | 1.47E-06 | 9.40E-04 | D | 5.35E-02 | D; C | D; C |
| rs9261083 | 6 | 29969205 | 2.87E-06 | D; C | 1.47E-06 | 9.40E-04 | D | 5.35E-02 | D; C | D; C |
| rs9261364 | 6 | 30056985 | 2.87E-06 | D; C | 1.47E-06 | 9.40E-04 | D | 5.35E-02 | D; C | D; C |
| rs77713789 | 6 | 57591378 | 2.97E-06 | C; M | 2.75E-07 | 3.32E-04 | M | 1.65E-01 | C; M | C; M |
| rs9261276 | 6 | 30031059 | 2.99E-06 | D; C | 4.88E-07 | 2.74E-03 | D | 2.57E-01 | D; C | D; C |
| rs1051133 | 6 | 30037908 | 3.09E-06 | D; C | 8.25E-07 | 2.17E-03 | D | 9.09E-02 | D; C | D; C |
| rs3807030 | 6 | 30033690 | 3.16E-06 | D; C | 5.85E-07 | 2.85E-03 | D | 2.50E-01 | D; C | D; C |
| rs9261080 | 6 | 29968987 | 3.22E-06 | D; C | 5.84E-07 | 2.95E-03 | D | 2.52E-01 | D; C | D; C |
| rs9261193 | 6 | 30002173 | 3.23E-06 | D; C | 6.62E-07 | 3.07E-03 | D | 2.47E-01 | D; C | D; C |
| rs9261041 | 6 | 29966718 | 3.29E-06 | D; C | 5.99E-07 | 2.80E-03 | D | 2.29E-01 | D; C | D; C |
| rs9261160 | 6 | 29993086 | 3.30E-06 | D; C | 7.98E-07 | 3.17E-03 | D | 2.64E-01 | D; C | D; C |
| rs16914571 | 10 | 61793446 | 3.32E-06 | C; M | 4.11E-07 | 1.17E-02 | C | 1.46E-01 | C; M | C; M |
| rs9261090 | 6 | 29969929 | 3.32E-06 | D; C | 6.44E-07 | 2.97E-03 | D | 2.47E-01 | D; C | D; C |
| rs9261036 | 6 | 29966301 | 3.39E-06 | D; C | 7.16E-07 | 3.02E-03 | D | 2.55E-01 | D; C | D; C |
| rs3734836 | 6 | 29980073 | 3.40E-06 | D; C | 7.25E-07 | 3.11E-03 | D | 2.52E-01 | D; C | D; C |
| rs3823372 | 6 | 29943981 | 3.40E-06 | D; C | 6.28E-07 | 2.79E-03 | D | 3.46E-01 | D; C | D; C |
| rs9261151 | 6 | 29987738 | 3.43E-06 | D; C | 7.27E-07 | 3.02E-03 | D | 2.55E-01 | D; C | D; C |
| rs9260952 | 6 | 29959746 | 3.45E-06 | D; C | 8.83E-07 | 2.56E-03 | D | 2.49E-01 | D; C | D; C |
| rs10745 | 6 | 30030057 | 3.47E-06 | D; C | 6.89E-07 | 2.98E-03 | D | 2.54E-01 | D; C | D; C |
| rs2240067 | 6 | 30037659 | 3.47E-06 | D; C | 6.89E-07 | 2.98E-03 | D | 2.54E-01 | D; C | D; C |
| rs2301751 | 6 | 30039484 | 3.47E-06 | D; C | 6.89E-07 | 2.98E-03 | D | 2.54E-01 | D; C | D; C |
| rs3757332 | 6 | 30028477 | 3.47E-06 | D; C | 6.89E-07 | 2.98E-03 | D | 2.54E-01 | D; C | D; C |
| rs3807036 | 6 | 30044914 | 3.47E-06 | D; C | 6.89E-07 | 2.98E-03 | D | 2.54E-01 | D; C | D; C |
| rs3891156 | 6 | 29988740 | 3.47E-06 | D; C | 6.89E-07 | 2.98E-03 | D | 2.54E-01 | D; C | D; C |
| rs3891157 | 6 | 29988439 | 3.47E-06 | D; C | 6.89E-07 | 2.98E-03 | D | 2.54E-01 | D; C | D; C |
| rs4993665 | 6 | 29784876 | 3.47E-06 | D; C | 6.89E-07 | 2.98E-03 | D | 2.54E-01 | D; C | D; C |
| rs6903621 | 6 | 29967486 | 3.47E-06 | D; C | 6.89E-07 | 2.98E-03 | D | 2.54E-01 | D; C | D; C |
| rs6912454 | 6 | 29990044 | 3.47E-06 | D; C | 6.89E-07 | 2.98E-03 | D | 2.54E-01 | D; C | D; C |
| rs6923832 | 6 | 30062058 | 3.47E-06 | D; C | 6.89E-07 | 2.98E-03 | D | 2.54E-01 | D; C | D; C |
| rs6923856 | 6 | 29967529 | 3.47E-06 | D; C | 6.89E-07 | 2.98E-03 | D | 2.54E-01 | D; C | D; C |
| rs6925061 | 6 | 29992286 | 3.47E-06 | D; C | 6.89E-07 | 2.98E-03 | D | 2.54E-01 | D; C | D; C |
| rs7770505 | 6 | 30028913 | 3.47E-06 | D; C | 6.89E-07 | 2.98E-03 | D | 2.54E-01 | D; C | D; C |
| rs9260941 | 6 | 29958865 | 3.47E-06 | D; C | 6.89E-07 | 2.98E-03 | D | 2.54E-01 | D; C | D; C |
| rs9260946 | 6 | 29959254 | 3.47E-06 | D; C | 6.89E-07 | 2.98E-03 | D | 2.54E-01 | D; C | D; C |
| rs9260948 | 6 | 29959326 | 3.47E-06 | D; C | 6.89E-07 | 2.98E-03 | D | 2.54E-01 | D; C | D; C |
| rs9260954 | 6 | 29959935 | 3.47E-06 | D; C | 6.89E-07 | 2.98E-03 | D | 2.54E-01 | D; C | D; C |
| rs9260957 | 6 | 29960083 | 3.47E-06 | D; C | 6.89E-07 | 2.98E-03 | D | 2.54E-01 | D; C | D; C |
| rs9260984 | 6 | 29962268 | 3.47E-06 | D; C | 6.89E-07 | 2.98E-03 | D | 2.54E-01 | D; C | D; C |
| rs9260998 | 6 | 29963622 | 3.47E-06 | D; C | 6.89E-07 | 2.98E-03 | D | 2.54E-01 | D; C | D; C |
| rs9261029 | 6 | 29966041 | 3.47E-06 | D; C | 6.89E-07 | 2.98E-03 | D | 2.54E-01 | D; C | D; C |
| rs9261037 | 6 | 29966465 | 3.47E-06 | D; C | 6.89E-07 | 2.98E-03 | D | 2.54E-01 | D; C | D; C |
| rs9261040 | 6 | 29966620 | 3.47E-06 | D; C | 6.89E-07 | 2.98E-03 | D | 2.54E-01 | D; C | D; C |
| rs9261045 | 6 | 29966867 | 3.47E-06 | D; C | 6.89E-07 | 2.98E-03 | D | 2.54E-01 | D; C | D; C |
| rs9261081 | 6 | 29969040 | 3.47E-06 | D; C | 6.89E-07 | 2.98E-03 | D | 2.54E-01 | D; C | D; C |
| rs9261091 | 6 | 29969973 | 3.47E-06 | D; C | 6.89E-07 | 2.98E-03 | D | 2.54E-01 | D; C | D; C |
| rs9261093 | 6 | 29970685 | 3.47E-06 | D; C | 6.89E-07 | 2.98E-03 | D | 2.54E-01 | D; C | D; C |
| rs9261094 | 6 | 29970839 | 3.47E-06 | D; C | 6.89E-07 | 2.98E-03 | D | 2.54E-01 | D; C | D; C |
| rs9261108 | 6 | 29975587 | 3.47E-06 | D; C | 6.89E-07 | 2.98E-03 | D | 2.54E-01 | D; C | D; C |
| rs9261118 | 6 | 29979125 | 3.47E-06 | D; C | 6.89E-07 | 2.98E-03 | D | 2.54E-01 | D; C | D; C |
| rs9261125 | 6 | 29979375 | 3.47E-06 | D; C | 6.89E-07 | 2.98E-03 | D | 2.54E-01 | D; C | D; C |
| rs9261130 | 6 | 29980445 | 3.47E-06 | D; C | 6.89E-07 | 2.98E-03 | D | 2.54E-01 | D; C | D; C |
| rs9261133 | 6 | 29981187 | 3.47E-06 | D; C | 6.89E-07 | 2.98E-03 | D | 2.54E-01 | D; C | D; C |
| rs9261138 | 6 | 29981515 | 3.47E-06 | D; C | 6.89E-07 | 2.98E-03 | D | 2.54E-01 | D; C | D; C |
| rs9261156 | 6 | 29991538 | 3.47E-06 | D; C | 6.89E-07 | 2.98E-03 | D | 2.54E-01 | D; C | D; C |
| rs9261158 | 6 | 29992953 | 3.47E-06 | D; C | 6.89E-07 | 2.98E-03 | D | 2.54E-01 | D; C | D; C |
| rs9261159 | 6 | 29992973 | 3.47E-06 | D; C | 6.89E-07 | 2.98E-03 | D | 2.54E-01 | D; C | D; C |
| rs9261171 | 6 | 29996121 | 3.47E-06 | D; C | 6.89E-07 | 2.98E-03 | D | 2.54E-01 | D; C | D; C |
| rs9261191 | 6 | 30001950 | 3.47E-06 | D; C | 6.89E-07 | 2.98E-03 | D | 2.54E-01 | D; C | D; C |
| rs9261203 | 6 | 30005043 | 3.47E-06 | D; C | 6.89E-07 | 2.98E-03 | D | 2.54E-01 | D; C | D; C |
| rs9261218 | 6 | 30011275 | 3.47E-06 | D; C | 6.89E-07 | 2.98E-03 | D | 2.54E-01 | D; C | D; C |
| rs9261219 | 6 | 30011451 | 3.47E-06 | D; C | 6.89E-07 | 2.98E-03 | D | 2.54E-01 | D; C | D; C |
| rs9261220 | 6 | 30011863 | 3.47E-06 | D; C | 6.89E-07 | 2.98E-03 | D | 2.54E-01 | D; C | D; C |
| rs9261257 | 6 | 30022425 | 3.47E-06 | D; C | 6.89E-07 | 2.98E-03 | D | 2.54E-01 | D; C | D; C |
| rs9261263 | 6 | 30024749 | 3.47E-06 | D; C | 6.89E-07 | 2.98E-03 | D | 2.54E-01 | D; C | D; C |
| rs9261265 | 6 | 30026350 | 3.47E-06 | D; C | 6.89E-07 | 2.98E-03 | D | 2.54E-01 | D; C | D; C |
| rs9261270 | 6 | 30030135 | 3.47E-06 | D; C | 6.89E-07 | 2.98E-03 | D | 2.54E-01 | D; C | D; C |
| rs9261316 | 6 | 30047837 | 3.47E-06 | D; C | 6.89E-07 | 2.98E-03 | D | 2.54E-01 | D; C | D; C |
| rs9261317 | 6 | 30048305 | 3.47E-06 | D; C | 6.89E-07 | 2.98E-03 | D | 2.54E-01 | D; C | D; C |
| rs9261360 | 6 | 30055667 | 3.47E-06 | D; C | 6.89E-07 | 2.98E-03 | D | 2.54E-01 | D; C | D; C |
| rs9261365 | 6 | 30057675 | 3.47E-06 | D; C | 6.89E-07 | 2.98E-03 | D | 2.54E-01 | D; C | D; C |
| rs9261372 | 6 | 30059055 | 3.47E-06 | D; C | 6.89E-07 | 2.98E-03 | D | 2.54E-01 | D; C | D; C |
| rs9261375 | 6 | 30059451 | 3.47E-06 | D; C | 6.89E-07 | 2.98E-03 | D | 2.54E-01 | D; C | D; C |
| rs9261386 | 6 | 30061293 | 3.47E-06 | D; C | 6.89E-07 | 2.98E-03 | D | 2.54E-01 | D; C | D; C |
| rs6940082 | 6 | 29975095 | 3.48E-06 | D; C | 7.25E-07 | 2.94E-03 | D | 2.58E-01 | D; C | D; C |
| rs10994154 | 10 | 61789753 | 3.50E-06 | C; M | 4.06E-07 | 1.10E-02 | C | 1.48E-01 | C; M | C; M |
| rs9261095 | 6 | 29971014 | 3.54E-06 | D; C | 4.92E-07 | 2.74E-03 | D | 2.47E-01 | D; C | D; C |
| rs9261207 | 6 | 30006482 | 3.65E-06 | D; C | 7.43E-07 | 3.06E-03 | D | 2.52E-01 | D; C | D; C |
| rs3893185 | 4 | 6874834 | 3.69E-06 | M | 5.29E-05 | 8.43E-02 | M | - | - | M |
| rs9261271 | 6 | 30030189 | 3.73E-06 | D; C | 7.76E-07 | 3.21E-03 | D | 2.66E-01 | D; C | D; C |
| rs7771672 | 6 | 30016694 | 3.79E-06 | D; C | 8.15E-07 | 3.14E-03 | D | 2.65E-01 | D; C | D; C |
| rs9261044 | 6 | 29966749 | 3.82E-06 | D; C | 6.34E-07 | 2.90E-03 | D | 2.59E-01 | D; C | D; C |
| rs379192 | 19 | 56570197 | 3.94E-06 | D; C | 4.97E-07 | 1.06E-03 | C | 9.62E-01 | D; C | D; C |
| rs437392 | 19 | 56574052 | 3.94E-06 | D; C | 4.97E-07 | 1.06E-03 | C | 9.62E-01 | D; C | D; C |
| rs9260932 | 6 | 29957802 | 3.96E-06 | D; C | 5.98E-07 | 3.61E-03 | D | 2.14E-01 | D; C | D; C |
| rs9261361 | 6 | 30055933 | 3.97E-06 | D; C | 7.84E-07 | 3.19E-03 | D | 2.43E-01 | D; C | D; C |
| rs7771476 | 6 | 30016573 | 4.03E-06 | D; C | 7.83E-07 | 3.24E-03 | D | 2.58E-01 | D; C | D; C |
| rs6919438 | 6 | 29991649 | 4.42E-06 | D; C | 1.01E-06 | 3.58E-03 | D | 2.54E-01 | D; C | D; C |
| rs10902227 | 11 | 845789 | 4.62E-06 | C | 2.39E-05 | 9.86E-01 | C | - | - | C |
| rs3091238 | 19 | 53783181 | 5.47E-06 | C | 1.21E-05 | 9.77E-02 | C | - | - | C |
| rs3113752 | 4 | 181519492 | 5.48E-06 | M | 1.24E-05 | 2.90E-01 | M | - | - | M |
| rs9259705 | 6 | 29881038 | 5.71E-06 | D; C | 5.55E-06 | 9.73E-04 | D | 1.66E-01 | D; C | D; C |
| rs9260920 | 6 | 29949047 | 5.76E-06 | D; C | 8.57E-07 | 1.13E-03 | D | 2.10E-01 | D; C | D; C |
| rs17860779 | 6 | 170858332 | 5.92E-06 | D; M | 7.79E-07 | 1.20E-03 | M | 7.55E-01 | D; M | D; M |
| rs10967764 | 9 | 27189869 | 5.94E-06 | D; C; M | 4.71E-08 | 6.19E-04 | M | 3.84E-02 | C; M | D; C; M |
| rs9460196 | 6 | 170783347 | 6.00E-06 | D; M | 7.41E-07 | 1.20E-03 | M | 7.58E-01 | D; M | D; M |
| rs994346 | 4 | 181523057 | 6.56E-06 | M | 6.17E-05 | 8.05E-02 | M | - | - | M |
| rs6845985 | 4 | 6855767 | 7.02E-06 | M | 5.42E-05 | 1.17E-01 | M | - | - | M |
| rs7395060 | 11 | 119703617 | 7.11E-06 | D; C; M | 2.17E-06 | 1.41E-03 | D | 3.48E-02 | D; M | D; C; M |
| rs4819806 | 22 | 19540361 | 7.30E-06 | C | 2.10E-06 | 1.57E-01 | C | - | - | C |
| rs7912984 | 10 | 70343285 | 7.30E-06 | C; M | 5.22E-04 | 4.07E-02 | C | 6.60E-01 | C; M | C; M |
| rs2865243 | 19 | 53787841 | 7.37E-06 | C | 2.78E-05 | 7.84E-02 | C | - | - | C |
| rs12854887 | 23 | 51338769 | 7.78E-06 | D | 5.96E-06 | 2.72E-01 | D | - | - | D |
| rs1858608 | 10 | 95970288 | 8.00E-06 | C | 2.16E-06 | 3.93E-01 | C | - | - | C |
| rs75337932 | 4 | 188968298 | 8.01E-06 | C; M | 1.46E-05 | 4.42E-02 | M | 2.77E-01 | C; M | C; M |
| rs9261096 | 6 | 29971039 | 8.31E-06 | D; C | 1.79E-06 | 5.12E-04 | D | 7.58E-01 | D; C | D; C |
| rs10823231 | 10 | 70338978 | 8.31E-06 | C; M | 6.93E-04 | 4.85E-02 | C | 6.61E-01 | C; M | C; M |
| rs35064938 | 23 | 51331771 | 8.48E-06 | D | 6.48E-06 | 2.68E-01 | D | - | - | D |
| rs3960965 | 19 | 53789383 | 8.52E-06 | C; M | 2.37E-05 | 4.49E-02 | C | 3.47E-01 | C; M | C; M |
| rs2488047 | 10 | 70371743 | 8.78E-06 | C; M | 1.88E-04 | 1.95E-03 | D | 6.03E-02 | C; M | C; M |
| rs12850240 | 23 | 51292502 | 9.26E-06 | D | 6.89E-06 | 2.71E-01 | D | - | - | D |
| rs78309007 | 4 | 7097324 | 9.33E-06 | C; M | 1.13E-06 | 1.68E-02 | M | 5.54E-02 | C; M | C; M |
| rs13209442 | 6 | 24597176 | 9.37E-06 | C | 3.98E-06 | 7.68E-01 | C | - | - | C |
| rs12479371 | 2 | 139465016 | 9.37E-06 | D; M | 2.35E-05 | 6.01E-03 | M | 1.40E-01 | D; M | D; M |
| rs11497984 | 10 | 70337170 | 9.48E-06 | C; M | 6.80E-04 | 4.63E-02 | C | 6.48E-01 | C; M | C; M |
| rs7894063 | 10 | 70349116 | 9.48E-06 | C; M | 6.80E-04 | 4.63E-02 | C | 6.48E-01 | C; M | C; M |
| rs2106136 | 22 | 19547879 | 9.67E-06 | C | 2.88E-06 | 1.90E-01 | C | - | - | C |
| rs9260736 | 6 | 29932809 | 9.68E-06 | D; C | 4.03E-06 | 1.09E-02 | D | 1.52E-01 | D; C | D; C |
| rs12371702 | 12 | 13855377 | 9.83E-06 | D; M | 4.92E-06 | 7.69E-03 | M | 8.71E-02 | D; M | D; M |

SNP, nucleotide polymorphism; Chr, chromosome; BP, base pair; D, depression; C, cognition; M, memory.

^a^ The *P* value was derived from multivariate GWAS.

^b^ Sequential tests of pleiotropy with a *P* threshold of 0.05.

^c^ Single test of the number of phenotypes associated with genotype, H0 (test 0): all betas = 0.

^d^ Single test of the number of phenotypes associated with genotype, H0 (test 1): one or less beta is nonzero.

^e^ Single test of the number of phenotypes associated with genotype, H0 (test 2): two or less betas are nonzero.
