# Supplementary material for: Multivariate genome-wide association study of depression, cognition, and memory phenotypes and validation analysis identify 12 cross-ethnic variants
Source: Transl Psychiatry. 2022 Jul 30;12:304. doi: 10.1038/s41398-022-02074-x (PMC9338946; doi:10.1038/s41398-022-02074-x)
Supplement: Supplementary file 3 — Supplementary Table 3 [file 41398_2022_2074_MOESM3_ESM.doc]

**Supplementary Table 3** The enhancer enrichment results of top 100 depression-cognition-memory-related SNPs

| Tissues and cells | Observed | Expected (all SNPs) | Expected (GWAS SNPs) | Binomial *P* (all SNPs) | Binomial *P* (GWAS SNPs) |
| --- | --- | --- | --- | --- | --- |
| E087 PANC.ISLT (Pancreatic Islets) | 9 | 1.3 | 2 | 5.00E-06 | 1.78E-04 |
| E110 GI.STMC.MUC (Stomach Mucosa) | 14 | 3.5 | 6.1 | 1.10E-05 | 2.95E-03 |
| E084 GI.L.INT.FET (Fetal Intestine Large) | 14 | 3.6 | 6.6 | 1.60E-05 | 6.06E-03 |
| E046 BLD.CD56.PC (Primary Natural Killer cells from peripheral blood) | 12 | 3.4 | 6 | 1.73E-04 | 1.62E-02 |
| E044 BLD.CD4.CD25.CD127M.TREGPC (Primary T regulatory cells from peripheral blood) | 8 | 2 | 3.6 | 8.54E-04 | 2.88E-02 |
| E066 LIV.ADLT (Liver) | 12 | 3.9 | 6.9 | 4.94E-04 | 4.27E-02 |

SNP, nucleotide polymorphism.
