# Supplementary material for: Multivariate genome-wide association study of depression, cognition, and memory phenotypes and validation analysis identify 12 cross-ethnic variants
Source: Transl Psychiatry. 2022 Jul 30;12:304. doi: 10.1038/s41398-022-02074-x (PMC9338946; doi:10.1038/s41398-022-02074-x)
Supplement: Supplementary file 4 — Supplementary Table 4 [file 41398_2022_2074_MOESM4_ESM.doc]

**Supplementary Table 4** The results of pleiotropy analysis for multivariate GWAS of depression-cognition-memory identified SNPs (*P*<1×10^-5^) after imputation

| SNP | Chr | BP | *P* value ^a^ | Trait of nonzero *β* ^b^ | *P* for test 0 ^c^ | *P* for test 1 ^d^ | Trait of nonzero *β* for test 1 ^d^ | *P* for test 2 ^e^ | Trait of nonzero *β* for test 2 ^e^ | Associated phenotype |
| --- | --- | --- | --- | --- | --- | --- | --- | --- | --- | --- |
| chr6:24597173 | 6 | 24597173 | 1.71E-07 | C | 1.48E-07 | 5.25E-01 | C | - | - | C |
| rs12210323 | 6 | 24591728 | 1.71E-07 | C | 1.48E-07 | 5.25E-01 | C | - | - | C |
| rs12213116 | 6 | 24594630 | 1.72E-07 | C | 1.55E-07 | 5.26E-01 | C | - | - | C |
| rs61783213 | 1 | 77260333 | 2.77E-07 | C; M | 1.14E-09 | 1.91E-05 | C | 1.00 | C; M | C; M |
| rs9589468 | 13 | 92916268 | 3.25E-07 | D; C; M | 1.52E-07 | 2.62E-04 | D | 1.17E-02 | D; C | D; C; M |
| rs67007022 | 4 | 181525540 | 3.46E-07 | M | 2.15E-05 | 1.64E-01 | M | - | - | M |
| rs533752605 | 3 | 84415473 | 3.55E-07 | D; C | 4.77E-09 | 1.03E-04 | C | 1.55E-01 | D; C | D; C |
| rs11577464 | 1 | 77259107 | 5.71E-07 | C; M | 4.81E-09 | 2.07E-05 | C | 8.29E-01 | C; M | C; M |
| rs112897238 | 1 | 77260102 | 6.31E-07 | C; M | 2.45E-09 | 2.49E-05 | C | 9.32E-01 | C; M | C; M |
| rs73198369 | 4 | 60865968 | 6.93E-07 | M | 7.09E-07 | 2.88E-01 | M | - | - | M |
| rs374850664 | 10 | 70336105 | 7.36E-07 | C; M | 7.38E-05 | 1.50E-02 | C | 5.34E-01 | C; M | C; M |
| rs8036389 | 15 | 97492430 | 7.46E-07 | D | 2.11E-04 | 1.44E-01 | D | - | - | D |
| rs11599886 | 10 | 70324991 | 8.42E-07 | C | 3.38E-04 | 6.45E-02 | C | - | - | C |
| rs7898805 | 10 | 120566433 | 8.54E-07 | D; C | 3.29E-06 | 8.89E-04 | C | 3.90E-01 | D; C | D; C |
| rs58350164 | 10 | 120568419 | 8.99E-07 | D; C | 2.84E-07 | 3.11E-04 | D | 2.00E-01 | D; C | D; C |
| rs61012528 | 10 | 120568716 | 9.79E-07 | D; C | 2.15E-06 | 7.31E-04 | C | 3.83E-01 | D; C | D; C |
| rs9589470 | 13 | 92917856 | 1.18E-06 | D; C; M | 7.07E-07 | 1.64E-03 | D | 2.71E-02 | D; C | D; C; M |
| rs117254295 | 10 | 120563039 | 1.30E-06 | D; C | 1.95E-06 | 1.86E-03 | C | 6.16E-01 | D; C | D; C |
| rs34631623 | 18 | 29438021 | 1.38E-06 | D; C | 1.00E-04 | 1.09E-02 | D | 6.06E-01 | D; C | D; C |
| rs79882698 | 10 | 120569407 | 1.41E-06 | D; C | 3.36E-06 | 1.45E-03 | C | 4.00E-01 | D; C | D; C |
| rs200223722 | 23 | 113705416 | 1.41E-06 | D | 4.28E-06 | 1.47E-01 | D | - | - | D |
| rs3967317 | 3 | 3058707 | 1.42E-06 | D; M | 5.12E-07 | 8.81E-03 | M | 2.89E-01 | D; M | D; M |
| rs9685974 | 4 | 6869435 | 1.50E-06 | M | 1.35E-05 | 1.18E-01 | M | - | - | M |
| rs149856258 | 20 | 13823497 | 1.59E-06 | D; C; M | 1.53E-06 | 3.66E-03 | M | 3.89E-02 | D; M | D; C; M |
| rs77309903 | 5 | 115296336 | 1.75E-06 | C | 1.51E-06 | 6.15E-02 | C | - | - | C |
| rs114941840 | 19 | 22443558 | 1.76E-06 | C; M | 5.81E-06 | 4.77E-04 | M | 4.03E-01 | C; M | C; M |
| rs141516760 | 5 | 115292748 | 1.76E-06 | C | 1.65E-06 | 6.03E-02 | C | - | - | C |
| rs145616421 | 5 | 115288229 | 1.76E-06 | C | 1.65E-06 | 6.03E-02 | C | - | - | C |
| rs74645605 | 5 | 115293587 | 1.76E-06 | C | 1.65E-06 | 6.03E-02 | C | - | - | C |
| rs200306190 | 10 | 120566065 | 1.84E-06 | D; C | 3.34E-06 | 2.11E-03 | C | 5.50E-01 | D; C | D; C |
| rs7087686 | 10 | 120569141 | 1.93E-06 | D; C | 2.90E-06 | 9.58E-04 | C | 3.26E-01 | D; C | D; C |
| rs9260918 | 6 | 29948751 | 2.00E-06 | D; C | 5.76E-07 | 2.41E-03 | D | 7.55E-02 | D; C | D; C |
| rs4985694 | 17 | 16861332 | 2.29E-06 | C | 5.37E-07 | 1.86E-01 | C | - | - | C |
| rs67361271 | 10 | 70325233 | 2.38E-06 | C; M | 3.61E-04 | 1.11E-02 | C | 4.38E-01 | C; M | C; M |
| rs150994751 | 6 | 30049879 | 2.40E-06 | D; C | 5.03E-07 | 9.63E-04 | D | 2.42E-01 | D; C | D; C |
| rs199649857 | 6 | 30049185 | 2.40E-06 | D; C | 5.03E-07 | 9.63E-04 | D | 2.42E-01 | D; C | D; C |
| rs199924226 | 6 | 30050931 | 2.40E-06 | D; C | 5.03E-07 | 9.63E-04 | D | 2.42E-01 | D; C | D; C |
| rs200404637 | 6 | 30049500 | 2.40E-06 | D; C | 5.03E-07 | 9.63E-04 | D | 2.42E-01 | D; C | D; C |
| rs200957442 | 6 | 30050013 | 2.40E-06 | D; C | 5.03E-07 | 9.63E-04 | D | 2.42E-01 | D; C | D; C |
| rs1331848 | 13 | 31508724 | 2.43E-06 | D; C; M | 2.35E-08 | 1.46E-05 | C | 4.86E-03 | C; M | D; C; M |
| rs2522110 | 17 | 34485994 | 2.50E-06 | D; C | 2.88E-06 | 3.87E-03 | D | 4.41E-01 | D; C | D; C |
| rs5919909 | 23 | 144910142 | 2.57E-06 | D; C | 3.88E-06 | 2.34E-02 | D | 1.78E-01 | D; C | D; C |
| rs2041433 | 15 | 97493539 | 2.63E-06 | D | 9.73E-05 | 3.14E-01 | D | - | - | D |
| rs6456223 | 6 | 170790632 | 2.67E-06 | D; M | 2.74E-05 | 2.13E-03 | M | 5.41E-01 | D; M | D; M |
| rs112331160 | 6 | 29944721 | 2.71E-06 | D; C | 5.90E-07 | 3.79E-03 | D | 2.17E-01 | D; C | D; C |
| rs2232239 | 6 | 29942866 | 2.71E-06 | D; C | 5.90E-07 | 3.79E-03 | D | 2.17E-01 | D; C | D; C |
| rs2894000 | 6 | 29944666 | 2.71E-06 | D; C | 5.90E-07 | 3.79E-03 | D | 2.17E-01 | D; C | D; C |
| rs369678743 | 6 | 29933954 | 2.71E-06 | D; C | 5.90E-07 | 3.79E-03 | D | 2.17E-01 | D; C | D; C |
| rs3734832 | 6 | 29950492 | 2.71E-06 | D; C | 5.90E-07 | 3.79E-03 | D | 2.17E-01 | D; C | D; C |
| rs3823364 | 6 | 29943802 | 2.71E-06 | D; C | 5.90E-07 | 3.79E-03 | D | 2.17E-01 | D; C | D; C |
| rs3823372 | 6 | 29943981 | 2.71E-06 | D; C | 5.90E-07 | 3.79E-03 | D | 2.17E-01 | D; C | D; C |
| rs3823382 | 6 | 29945210 | 2.71E-06 | D; C | 5.90E-07 | 3.79E-03 | D | 2.17E-01 | D; C | D; C |
| rs3823384 | 6 | 29945368 | 2.71E-06 | D; C | 5.90E-07 | 3.79E-03 | D | 2.17E-01 | D; C | D; C |
| rs3901554 | 6 | 29955025 | 2.71E-06 | D; C | 5.90E-07 | 3.79E-03 | D | 2.17E-01 | D; C | D; C |
| rs9260717 | 6 | 29931376 | 2.71E-06 | D; C | 5.90E-07 | 3.79E-03 | D | 2.17E-01 | D; C | D; C |
| rs9260718 | 6 | 29931390 | 2.71E-06 | D; C | 5.90E-07 | 3.79E-03 | D | 2.17E-01 | D; C | D; C |
| rs9260720 | 6 | 29931419 | 2.71E-06 | D; C | 5.90E-07 | 3.79E-03 | D | 2.17E-01 | D; C | D; C |
| rs9260733 | 6 | 29932433 | 2.71E-06 | D; C | 5.90E-07 | 3.79E-03 | D | 2.17E-01 | D; C | D; C |
| rs9260736 | 6 | 29932809 | 2.71E-06 | D; C | 5.90E-07 | 3.79E-03 | D | 2.17E-01 | D; C | D; C |
| rs9260768 | 6 | 29937304 | 2.71E-06 | D; C | 5.90E-07 | 3.79E-03 | D | 2.17E-01 | D; C | D; C |
| rs9260824 | 6 | 29942439 | 2.71E-06 | D; C | 5.90E-07 | 3.79E-03 | D | 2.17E-01 | D; C | D; C |
| rs9260828 | 6 | 29942678 | 2.71E-06 | D; C | 5.90E-07 | 3.79E-03 | D | 2.17E-01 | D; C | D; C |
| rs9260830 | 6 | 29942979 | 2.71E-06 | D; C | 5.90E-07 | 3.79E-03 | D | 2.17E-01 | D; C | D; C |
| rs9260836 | 6 | 29943528 | 2.71E-06 | D; C | 5.90E-07 | 3.79E-03 | D | 2.17E-01 | D; C | D; C |
| rs9260837 | 6 | 29943581 | 2.71E-06 | D; C | 5.90E-07 | 3.79E-03 | D | 2.17E-01 | D; C | D; C |
| rs9260859 | 6 | 29944931 | 2.71E-06 | D; C | 5.90E-07 | 3.79E-03 | D | 2.17E-01 | D; C | D; C |
| rs9260860 | 6 | 29945508 | 2.71E-06 | D; C | 5.90E-07 | 3.79E-03 | D | 2.17E-01 | D; C | D; C |
| rs9260861 | 6 | 29945594 | 2.71E-06 | D; C | 5.90E-07 | 3.79E-03 | D | 2.17E-01 | D; C | D; C |
| rs9260862 | 6 | 29945741 | 2.71E-06 | D; C | 5.90E-07 | 3.79E-03 | D | 2.17E-01 | D; C | D; C |
| rs9260863 | 6 | 29945771 | 2.71E-06 | D; C | 5.90E-07 | 3.79E-03 | D | 2.17E-01 | D; C | D; C |
| rs9260864 | 6 | 29945841 | 2.71E-06 | D; C | 5.90E-07 | 3.79E-03 | D | 2.17E-01 | D; C | D; C |
| rs9260865 | 6 | 29945898 | 2.71E-06 | D; C | 5.90E-07 | 3.79E-03 | D | 2.17E-01 | D; C | D; C |
| rs9260868 | 6 | 29946157 | 2.71E-06 | D; C | 5.90E-07 | 3.79E-03 | D | 2.17E-01 | D; C | D; C |
| rs9260869 | 6 | 29946453 | 2.71E-06 | D; C | 5.90E-07 | 3.79E-03 | D | 2.17E-01 | D; C | D; C |
| rs9260870 | 6 | 29946503 | 2.71E-06 | D; C | 5.90E-07 | 3.79E-03 | D | 2.17E-01 | D; C | D; C |
| rs9260871 | 6 | 29946571 | 2.71E-06 | D; C | 5.90E-07 | 3.79E-03 | D | 2.17E-01 | D; C | D; C |
| rs9260872 | 6 | 29946577 | 2.71E-06 | D; C | 5.90E-07 | 3.79E-03 | D | 2.17E-01 | D; C | D; C |
| rs9260873 | 6 | 29946656 | 2.71E-06 | D; C | 5.90E-07 | 3.79E-03 | D | 2.17E-01 | D; C | D; C |
| rs9260913 | 6 | 29948204 | 2.71E-06 | D; C | 5.90E-07 | 3.79E-03 | D | 2.17E-01 | D; C | D; C |
| rs9260914 | 6 | 29948401 | 2.71E-06 | D; C | 5.90E-07 | 3.79E-03 | D | 2.17E-01 | D; C | D; C |
| rs9260915 | 6 | 29948557 | 2.71E-06 | D; C | 5.90E-07 | 3.79E-03 | D | 2.17E-01 | D; C | D; C |
| rs9260916 | 6 | 29948708 | 2.71E-06 | D; C | 5.90E-07 | 3.79E-03 | D | 2.17E-01 | D; C | D; C |
| rs9260917 | 6 | 29948716 | 2.71E-06 | D; C | 5.90E-07 | 3.79E-03 | D | 2.17E-01 | D; C | D; C |
| rs9260919 | 6 | 29948884 | 2.71E-06 | D; C | 5.90E-07 | 3.79E-03 | D | 2.17E-01 | D; C | D; C |
| rs9260920 | 6 | 29949047 | 2.71E-06 | D; C | 5.90E-07 | 3.79E-03 | D | 2.17E-01 | D; C | D; C |
| rs9260921 | 6 | 29949091 | 2.71E-06 | D; C | 5.90E-07 | 3.79E-03 | D | 2.17E-01 | D; C | D; C |
| rs9260923 | 6 | 29949889 | 2.71E-06 | D; C | 5.90E-07 | 3.79E-03 | D | 2.17E-01 | D; C | D; C |
| rs9260929 | 6 | 29951782 | 2.71E-06 | D; C | 5.90E-07 | 3.79E-03 | D | 2.17E-01 | D; C | D; C |
| rs9260930 | 6 | 29956953 | 2.71E-06 | D; C | 5.90E-07 | 3.79E-03 | D | 2.17E-01 | D; C | D; C |
| rs34711016 | 4 | 181525603 | 2.87E-06 | D; M | 2.42E-05 | 1.50E-02 | M | 9.43E-01 | D; M | D; M |
| rs1245367 | 6 | 30053983 | 2.89E-06 | D; C | 5.61E-07 | 2.83E-03 | D | 2.58E-01 | D; C | D; C |
| rs1051133 | 6 | 30037908 | 2.90E-06 | D; C | 7.02E-07 | 2.18E-03 | D | 8.97E-02 | D; C | D; C |
| rs9260866 | 6 | 29946019 | 2.92E-06 | D; C | 6.40E-07 | 3.58E-03 | D | 2.08E-01 | D; C | D; C |
| rs138940267 | 6 | 30030927 | 2.94E-06 | D; C | 1.57E-06 | 9.84E-04 | D | 5.53E-02 | D; C | D; C |
| rs77713789 | 6 | 57591378 | 3.00E-06 | M | 4.54E-03 | 5.88E-02 | M | - | - | M |
| rs12663921 | 6 | 170782090 | 3.09E-06 | D; M | 7.41E-07 | 1.20E-03 | M | 7.58E-01 | D; M | D; M |
| rs35504837 | 6 | 170782041 | 3.09E-06 | D; M | 7.41E-07 | 1.20E-03 | M | 7.58E-01 | D; M | D; M |
| rs7682699 | 4 | 6854709 | 3.12E-06 | M | 1.88E-05 | 2.03E-01 | M | - | - | M |
| rs10745 | 6 | 30030057 | 3.22E-06 | D; C | 7.03E-07 | 3.08E-03 | D | 2.54E-01 | D; C | D; C |
| rs113615170 | 6 | 30022202 | 3.22E-06 | D; C | 7.03E-07 | 3.08E-03 | D | 2.54E-01 | D; C | D; C |
| rs370725085 | 6 | 30034343 | 3.22E-06 | D; C | 7.03E-07 | 3.08E-03 | D | 2.54E-01 | D; C | D; C |
| rs3757332 | 6 | 30028477 | 3.22E-06 | D; C | 7.03E-07 | 3.08E-03 | D | 2.54E-01 | D; C | D; C |
| rs3807030 | 6 | 30033690 | 3.22E-06 | D; C | 7.03E-07 | 3.08E-03 | D | 2.54E-01 | D; C | D; C |
| rs7770505 | 6 | 30028913 | 3.22E-06 | D; C | 7.03E-07 | 3.08E-03 | D | 2.54E-01 | D; C | D; C |
| rs9256991 | 6 | 30022203 | 3.22E-06 | D; C | 7.03E-07 | 3.08E-03 | D | 2.54E-01 | D; C | D; C |
| rs9261248 | 6 | 30019515 | 3.22E-06 | D; C | 7.03E-07 | 3.08E-03 | D | 2.54E-01 | D; C | D; C |
| rs9261250 | 6 | 30019654 | 3.22E-06 | D; C | 7.03E-07 | 3.08E-03 | D | 2.54E-01 | D; C | D; C |
| rs9261253 | 6 | 30020642 | 3.22E-06 | D; C | 7.03E-07 | 3.08E-03 | D | 2.54E-01 | D; C | D; C |
| rs9261257 | 6 | 30022425 | 3.22E-06 | D; C | 7.03E-07 | 3.08E-03 | D | 2.54E-01 | D; C | D; C |
| rs9261263 | 6 | 30024749 | 3.22E-06 | D; C | 7.03E-07 | 3.08E-03 | D | 2.54E-01 | D; C | D; C |
| rs9261264 | 6 | 30025210 | 3.22E-06 | D; C | 7.03E-07 | 3.08E-03 | D | 2.54E-01 | D; C | D; C |
| rs9261265 | 6 | 30026350 | 3.22E-06 | D; C | 7.03E-07 | 3.08E-03 | D | 2.54E-01 | D; C | D; C |
| rs9261270 | 6 | 30030135 | 3.22E-06 | D; C | 7.03E-07 | 3.08E-03 | D | 2.54E-01 | D; C | D; C |
| rs9261271 | 6 | 30030189 | 3.22E-06 | D; C | 7.03E-07 | 3.08E-03 | D | 2.54E-01 | D; C | D; C |
| rs9261276 | 6 | 30031059 | 3.22E-06 | D; C | 7.03E-07 | 3.08E-03 | D | 2.54E-01 | D; C | D; C |
| rs9280878 | 6 | 30029172 | 3.22E-06 | D; C | 7.03E-07 | 3.08E-03 | D | 2.54E-01 | D; C | D; C |
| rs371793437 | 6 | 30012654 | 3.30E-06 | D; C | 6.67E-07 | 2.97E-03 | D | 2.47E-01 | D; C | D; C |
| rs7771476 | 6 | 30016573 | 3.30E-06 | D; C | 6.67E-07 | 2.97E-03 | D | 2.47E-01 | D; C | D; C |
| rs7771672 | 6 | 30016694 | 3.30E-06 | D; C | 6.67E-07 | 2.97E-03 | D | 2.47E-01 | D; C | D; C |
| rs9261218 | 6 | 30011275 | 3.30E-06 | D; C | 6.67E-07 | 2.97E-03 | D | 2.47E-01 | D; C | D; C |
| rs9261219 | 6 | 30011451 | 3.30E-06 | D; C | 6.67E-07 | 2.97E-03 | D | 2.47E-01 | D; C | D; C |
| rs9261220 | 6 | 30011863 | 3.30E-06 | D; C | 6.67E-07 | 2.97E-03 | D | 2.47E-01 | D; C | D; C |
| rs9261242 | 6 | 30017629 | 3.30E-06 | D; C | 6.67E-07 | 2.97E-03 | D | 2.47E-01 | D; C | D; C |
| rs9280859 | 6 | 30013393 | 3.30E-06 | D; C | 6.67E-07 | 2.97E-03 | D | 2.47E-01 | D; C | D; C |
| rs9280865 | 6 | 30018024 | 3.30E-06 | D; C | 6.67E-07 | 2.97E-03 | D | 2.47E-01 | D; C | D; C |
| rs1760611657 | 6 | 29958255 | 3.32E-06 | D; C | 6.66E-07 | 3.03E-03 | D | 2.52E-01 | D; C | D; C |
| rs796244507 | 6 | 29958358 | 3.32E-06 | D; C | 6.66E-07 | 3.03E-03 | D | 2.52E-01 | D; C | D; C |
| rs113855257 | 6 | 29959364 | 3.32E-06 | D; C | 6.66E-07 | 3.03E-03 | D | 2.52E-01 | D; C | D; C |
| rs202063251 | 6 | 29959366 | 3.32E-06 | D; C | 6.66E-07 | 3.03E-03 | D | 2.52E-01 | D; C | D; C |
| rs9260931 | 6 | 29957508 | 3.32E-06 | D; C | 5.90E-07 | 3.79E-03 | D | 2.17E-01 | D; C | D; C |
| rs9260932 | 6 | 29957802 | 3.32E-06 | D; C | 6.66E-07 | 3.03E-03 | D | 2.52E-01 | D; C | D; C |
| rs9260933 | 6 | 29957866 | 3.32E-06 | D; C | 6.66E-07 | 3.03E-03 | D | 2.52E-01 | D; C | D; C |
| rs9260935 | 6 | 29958488 | 3.32E-06 | D; C | 6.66E-07 | 3.03E-03 | D | 2.52E-01 | D; C | D; C |
| rs9260936 | 6 | 29958502 | 3.32E-06 | D; C | 6.66E-07 | 3.03E-03 | D | 2.52E-01 | D; C | D; C |
| rs9260938 | 6 | 29958648 | 3.32E-06 | D; C | 6.66E-07 | 3.03E-03 | D | 2.52E-01 | D; C | D; C |
| rs9260939 | 6 | 29958674 | 3.32E-06 | D; C | 6.66E-07 | 3.03E-03 | D | 2.52E-01 | D; C | D; C |
| rs9260941 | 6 | 29958865 | 3.32E-06 | D; C | 6.66E-07 | 3.03E-03 | D | 2.52E-01 | D; C | D; C |
| rs9260943 | 6 | 29958933 | 3.32E-06 | D; C | 6.66E-07 | 3.03E-03 | D | 2.52E-01 | D; C | D; C |
| rs9260945 | 6 | 29959236 | 3.32E-06 | D; C | 6.66E-07 | 3.03E-03 | D | 2.52E-01 | D; C | D; C |
| rs9260946 | 6 | 29959254 | 3.32E-06 | D; C | 6.66E-07 | 3.03E-03 | D | 2.52E-01 | D; C | D; C |
| rs9260948 | 6 | 29959326 | 3.32E-06 | D; C | 6.66E-07 | 3.03E-03 | D | 2.52E-01 | D; C | D; C |
| rs9260950 | 6 | 29959458 | 3.32E-06 | D; C | 6.66E-07 | 3.03E-03 | D | 2.52E-01 | D; C | D; C |
| rs9260954 | 6 | 29959935 | 3.32E-06 | D; C | 6.66E-07 | 3.03E-03 | D | 2.52E-01 | D; C | D; C |
| rs9260956 | 6 | 29960074 | 3.32E-06 | D; C | 6.66E-07 | 3.03E-03 | D | 2.52E-01 | D; C | D; C |
| rs9260957 | 6 | 29960083 | 3.32E-06 | D; C | 6.66E-07 | 3.03E-03 | D | 2.52E-01 | D; C | D; C |
| rs9260958 | 6 | 29960193 | 3.32E-06 | D; C | 6.66E-07 | 3.03E-03 | D | 2.52E-01 | D; C | D; C |
| rs9260672 | 6 | 29924996 | 3.33E-06 | D; C | 5.65E-07 | 3.95E-03 | D | 2.23E-01 | D; C | D; C |
| rs9260693 | 6 | 29927534 | 3.33E-06 | D; C | 5.65E-07 | 3.95E-03 | D | 2.23E-01 | D; C | D; C |
| rs7250500 | 19 | 53789853 | 3.35E-06 | C; M | 2.71E-06 | 1.78E-02 | C | 1.39E-01 | C; M | C; M |
| rs9261160 | 6 | 29993086 | 3.36E-06 | D; C | 7.27E-07 | 3.03E-03 | D | 2.53E-01 | D; C | D; C |
| rs111582818 | 19 | 53779768 | 3.37E-06 | D; C | 6.69E-05 | 5.69E-03 | C | 4.04E-01 | D; C | D; C |
| rs9260989 | 6 | 29962517 | 3.40E-06 | D; C | 7.21E-07 | 2.97E-03 | D | 2.55E-01 | D; C | D; C |
| rs9260976 | 6 | 29961770 | 3.40E-06 | D; C | 7.21E-07 | 2.97E-03 | D | 2.55E-01 | D; C | D; C |
| rs9260981 | 6 | 29962028 | 3.40E-06 | D; C | 7.21E-07 | 2.97E-03 | D | 2.55E-01 | D; C | D; C |
| rs9260983 | 6 | 29962139 | 3.40E-06 | D; C | 7.21E-07 | 2.97E-03 | D | 2.55E-01 | D; C | D; C |
| rs9260984 | 6 | 29962268 | 3.40E-06 | D; C | 7.21E-07 | 2.97E-03 | D | 2.55E-01 | D; C | D; C |
| rs9260986 | 6 | 29962364 | 3.40E-06 | D; C | 7.21E-07 | 2.97E-03 | D | 2.55E-01 | D; C | D; C |
| rs9260993 | 6 | 29962595 | 3.40E-06 | D; C | 7.21E-07 | 2.97E-03 | D | 2.55E-01 | D; C | D; C |
| rs9260998 | 6 | 29963622 | 3.40E-06 | D; C | 7.21E-07 | 2.97E-03 | D | 2.55E-01 | D; C | D; C |
| rs9261005 | 6 | 29964314 | 3.40E-06 | D; C | 7.21E-07 | 2.97E-03 | D | 2.55E-01 | D; C | D; C |
| rs9261014 | 6 | 29964999 | 3.40E-06 | D; C | 7.21E-07 | 2.97E-03 | D | 2.55E-01 | D; C | D; C |
| rs9261025 | 6 | 29965938 | 3.40E-06 | D; C | 7.21E-07 | 2.97E-03 | D | 2.55E-01 | D; C | D; C |
| rs111785008 | 6 | 30038366 | 3.41E-06 | D; C | 6.41E-07 | 3.11E-03 | D | 2.55E-01 | D; C | D; C |
| rs138821691 | 6 | 30054031 | 3.41E-06 | D; C | 6.41E-07 | 3.11E-03 | D | 2.55E-01 | D; C | D; C |
| rs2240067 | 6 | 30037659 | 3.41E-06 | D; C | 6.41E-07 | 3.11E-03 | D | 2.55E-01 | D; C | D; C |
| rs2301751 | 6 | 30039484 | 3.41E-06 | D; C | 6.41E-07 | 3.11E-03 | D | 2.55E-01 | D; C | D; C |
| rs2301752 | 6 | 30039418 | 3.41E-06 | D; C | 6.41E-07 | 3.11E-03 | D | 2.55E-01 | D; C | D; C |
| rs9261284 | 6 | 30035805 | 3.41E-06 | D; C | 6.41E-07 | 3.11E-03 | D | 2.55E-01 | D; C | D; C |
| rs9261285 | 6 | 30036083 | 3.41E-06 | D; C | 6.41E-07 | 3.11E-03 | D | 2.55E-01 | D; C | D; C |
| rs9261286 | 6 | 30036269 | 3.41E-06 | D; C | 6.41E-07 | 3.11E-03 | D | 2.55E-01 | D; C | D; C |
| rs9261287 | 6 | 30036570 | 3.41E-06 | D; C | 6.41E-07 | 3.11E-03 | D | 2.55E-01 | D; C | D; C |
| rs9261296 | 6 | 30039752 | 3.41E-06 | D; C | 6.41E-07 | 3.11E-03 | D | 2.55E-01 | D; C | D; C |
| rs9261303 | 6 | 30042668 | 3.41E-06 | D; C | 6.41E-07 | 3.11E-03 | D | 2.55E-01 | D; C | D; C |
| rs9261304 | 6 | 30043171 | 3.41E-06 | D; C | 6.41E-07 | 3.11E-03 | D | 2.55E-01 | D; C | D; C |
| rs9261305 | 6 | 30043764 | 3.41E-06 | D; C | 6.41E-07 | 3.11E-03 | D | 2.55E-01 | D; C | D; C |
| rs9261313 | 6 | 30046752 | 3.41E-06 | D; C | 6.41E-07 | 3.11E-03 | D | 2.55E-01 | D; C | D; C |
| rs9261314 | 6 | 30046983 | 3.41E-06 | D; C | 6.41E-07 | 3.11E-03 | D | 2.55E-01 | D; C | D; C |
| rs9261315 | 6 | 30047503 | 3.41E-06 | D; C | 6.41E-07 | 3.11E-03 | D | 2.55E-01 | D; C | D; C |
| rs9261316 | 6 | 30047837 | 3.41E-06 | D; C | 6.41E-07 | 3.11E-03 | D | 2.55E-01 | D; C | D; C |
| rs9261317 | 6 | 30048305 | 3.41E-06 | D; C | 6.41E-07 | 3.11E-03 | D | 2.55E-01 | D; C | D; C |
| rs9261319 | 6 | 30048645 | 3.41E-06 | D; C | 6.41E-07 | 3.11E-03 | D | 2.55E-01 | D; C | D; C |
| rs9261359 | 6 | 30054746 | 3.41E-06 | D; C | 6.41E-07 | 3.11E-03 | D | 2.55E-01 | D; C | D; C |
| rs9261361 | 6 | 30055933 | 3.41E-06 | D; C | 6.41E-07 | 3.11E-03 | D | 2.55E-01 | D; C | D; C |
| rs9261076 | 6 | 29968655 | 3.44E-06 | D; C | 7.15E-07 | 2.94E-03 | D | 2.53E-01 | D; C | D; C |
| rs4405661 | 19 | 53789873 | 3.44E-06 | C; M | 3.06E-06 | 2.25E-02 | C | 1.67E-01 | C; M | C; M |
| rs9261273 | 6 | 30030641 | 3.46E-06 | D; C | 7.26E-07 | 3.09E-03 | D | 2.61E-01 | D; C | D; C |
| rs4949747 | 1 | 77257353 | 3.51E-06 | C; M | 9.55E-09 | 3.88E-05 | C | 7.93E-01 | C; M | C; M |
| rs6939616 | 6 | 29975093 | 3.53E-06 | D; C | 6.86E-07 | 3.02E-03 | D | 2.44E-01 | D; C | D; C |
| rs6940082 | 6 | 29975095 | 3.53E-06 | D; C | 6.86E-07 | 3.02E-03 | D | 2.44E-01 | D; C | D; C |
| rs2107200 | 6 | 30060436 | 3.54E-06 | D; C | 6.89E-07 | 2.98E-03 | D | 2.54E-01 | D; C | D; C |
| rs2107201 | 6 | 30060425 | 3.54E-06 | D; C | 6.89E-07 | 2.98E-03 | D | 2.54E-01 | D; C | D; C |
| rs2158288 | 6 | 30060255 | 3.54E-06 | D; C | 6.89E-07 | 2.98E-03 | D | 2.54E-01 | D; C | D; C |
| rs2158289 | 6 | 30060254 | 3.54E-06 | D; C | 6.89E-07 | 2.98E-03 | D | 2.54E-01 | D; C | D; C |
| rs2275853 | 6 | 29977068 | 3.54E-06 | D; C | 6.89E-07 | 2.98E-03 | D | 2.54E-01 | D; C | D; C |
| rs34422094 | 6 | 29973986 | 3.54E-06 | D; C | 6.89E-07 | 2.98E-03 | D | 2.54E-01 | D; C | D; C |
| rs368834955 | 6 | 30061047 | 3.54E-06 | D; C | 6.89E-07 | 2.98E-03 | D | 2.54E-01 | D; C | D; C |
| rs371236241 | 6 | 29973988 | 3.54E-06 | D; C | 6.89E-07 | 2.98E-03 | D | 2.54E-01 | D; C | D; C |
| rs6904969 | 6 | 29971431 | 3.54E-06 | D; C | 6.89E-07 | 2.98E-03 | D | 2.54E-01 | D; C | D; C |
| rs6923832 | 6 | 30062058 | 3.54E-06 | D; C | 6.89E-07 | 2.98E-03 | D | 2.54E-01 | D; C | D; C |
| rs6939480 | 6 | 29975055 | 3.54E-06 | D; C | 6.89E-07 | 2.98E-03 | D | 2.54E-01 | D; C | D; C |
| rs6939614 | 6 | 29975049 | 3.54E-06 | D; C | 6.89E-07 | 2.98E-03 | D | 2.54E-01 | D; C | D; C |
| rs76702759 | 6 | 30059451 | 3.54E-06 | D; C | 6.89E-07 | 2.98E-03 | D | 2.54E-01 | D; C | D; C |
| rs7750714 | 6 | 29974130 | 3.54E-06 | D; C | 6.89E-07 | 2.98E-03 | D | 2.54E-01 | D; C | D; C |
| rs77627704 | 6 | 30059273 | 3.54E-06 | D; C | 6.89E-07 | 2.98E-03 | D | 2.54E-01 | D; C | D; C |
| rs7768931 | 6 | 29974154 | 3.54E-06 | D; C | 6.89E-07 | 2.98E-03 | D | 2.54E-01 | D; C | D; C |
| rs9261095 | 6 | 29971014 | 3.54E-06 | D; C | 6.89E-07 | 2.98E-03 | D | 2.54E-01 | D; C | D; C |
| rs9261096 | 6 | 29971039 | 3.54E-06 | D; C | 6.89E-07 | 2.98E-03 | D | 2.54E-01 | D; C | D; C |
| rs9261099 | 6 | 29971913 | 3.54E-06 | D; C | 6.89E-07 | 2.98E-03 | D | 2.54E-01 | D; C | D; C |
| rs9261106 | 6 | 29974608 | 3.54E-06 | D; C | 6.89E-07 | 2.98E-03 | D | 2.54E-01 | D; C | D; C |
| rs9261107 | 6 | 29974672 | 3.54E-06 | D; C | 6.89E-07 | 2.98E-03 | D | 2.54E-01 | D; C | D; C |
| rs9261108 | 6 | 29975587 | 3.54E-06 | D; C | 6.89E-07 | 2.98E-03 | D | 2.54E-01 | D; C | D; C |
| rs9261109 | 6 | 29975834 | 3.54E-06 | D; C | 6.89E-07 | 2.98E-03 | D | 2.54E-01 | D; C | D; C |
| rs9261112 | 6 | 29976607 | 3.54E-06 | D; C | 6.89E-07 | 2.98E-03 | D | 2.54E-01 | D; C | D; C |
| rs9261114 | 6 | 29976857 | 3.54E-06 | D; C | 6.89E-07 | 2.98E-03 | D | 2.54E-01 | D; C | D; C |
| rs9261118 | 6 | 29979125 | 3.54E-06 | D; C | 6.89E-07 | 2.98E-03 | D | 2.54E-01 | D; C | D; C |
| rs9261191 | 6 | 30001950 | 3.54E-06 | D; C | 6.89E-07 | 2.98E-03 | D | 2.54E-01 | D; C | D; C |
| rs9261193 | 6 | 30002173 | 3.54E-06 | D; C | 6.89E-07 | 2.98E-03 | D | 2.54E-01 | D; C | D; C |
| rs9261203 | 6 | 30005043 | 3.54E-06 | D; C | 6.89E-07 | 2.98E-03 | D | 2.54E-01 | D; C | D; C |
| rs9261207 | 6 | 30006482 | 3.54E-06 | D; C | 6.89E-07 | 2.98E-03 | D | 2.54E-01 | D; C | D; C |
| rs9261213 | 6 | 30009214 | 3.54E-06 | D; C | 6.89E-07 | 2.98E-03 | D | 2.54E-01 | D; C | D; C |
| rs9261214 | 6 | 30009609 | 3.54E-06 | D; C | 6.89E-07 | 2.98E-03 | D | 2.54E-01 | D; C | D; C |
| rs9261362 | 6 | 30056177 | 3.54E-06 | D; C | 6.89E-07 | 2.98E-03 | D | 2.54E-01 | D; C | D; C |
| rs9261364 | 6 | 30056985 | 3.54E-06 | D; C | 6.89E-07 | 2.98E-03 | D | 2.54E-01 | D; C | D; C |
| rs9261365 | 6 | 30057675 | 3.54E-06 | D; C | 6.89E-07 | 2.98E-03 | D | 2.54E-01 | D; C | D; C |
| rs9261367 | 6 | 30058241 | 3.54E-06 | D; C | 6.89E-07 | 2.98E-03 | D | 2.54E-01 | D; C | D; C |
| rs9261369 | 6 | 30058605 | 3.54E-06 | D; C | 6.89E-07 | 2.98E-03 | D | 2.54E-01 | D; C | D; C |
| rs9261372 | 6 | 30059055 | 3.54E-06 | D; C | 6.89E-07 | 2.98E-03 | D | 2.54E-01 | D; C | D; C |
| rs9261377 | 6 | 30059819 | 3.54E-06 | D; C | 6.89E-07 | 2.98E-03 | D | 2.54E-01 | D; C | D; C |
| rs9261379 | 6 | 30059888 | 3.54E-06 | D; C | 6.89E-07 | 2.98E-03 | D | 2.54E-01 | D; C | D; C |
| rs9261381 | 6 | 30060002 | 3.54E-06 | D; C | 6.89E-07 | 2.98E-03 | D | 2.54E-01 | D; C | D; C |
| rs9261382 | 6 | 30060472 | 3.54E-06 | D; C | 6.89E-07 | 2.98E-03 | D | 2.54E-01 | D; C | D; C |
| rs9261386 | 6 | 30061293 | 3.54E-06 | D; C | 6.89E-07 | 2.98E-03 | D | 2.54E-01 | D; C | D; C |
| rs9278534 | 6 | 29971364 | 3.54E-06 | D; C | 6.89E-07 | 2.98E-03 | D | 2.54E-01 | D; C | D; C |
| rs9278546 | 6 | 30005761 | 3.54E-06 | D; C | 6.89E-07 | 2.98E-03 | D | 2.54E-01 | D; C | D; C |
| rs9280856 | 6 | 30004382 | 3.54E-06 | D; C | 6.89E-07 | 2.98E-03 | D | 2.54E-01 | D; C | D; C |
| rs6925856 | 6 | 30015069 | 3.55E-06 | D; C | 7.22E-07 | 2.82E-03 | D | 2.38E-01 | D; C | D; C |
| rs9261223 | 6 | 30013772 | 3.55E-06 | D; C | 7.22E-07 | 2.82E-03 | D | 2.38E-01 | D; C | D; C |
| rs9280861 | 6 | 30015502 | 3.55E-06 | D; C | 7.22E-07 | 2.82E-03 | D | 2.38E-01 | D; C | D; C |
| rs9261226 | 6 | 30014457 | 3.60E-06 | D; C | 7.08E-07 | 2.81E-03 | D | 2.41E-01 | D; C | D; C |
| rs796925533 | 6 | 29967002 | 3.62E-06 | D; C | 7.46E-07 | 2.92E-03 | D | 2.57E-01 | D; C | D; C |
| rs58226366 | 6 | 29967004 | 3.62E-06 | D; C | 7.46E-07 | 2.92E-03 | D | 2.57E-01 | D; C | D; C |
| rs201270778 | 6 | 29982015 | 3.62E-06 | D; C | 7.46E-07 | 2.92E-03 | D | 2.57E-01 | D; C | D; C |
| rs77798309 | 6 | 29990100 | 3.62E-06 | D; C | 7.46E-07 | 2.92E-03 | D | 2.57E-01 | D; C | D; C |
| rs113202273 | 6 | 29981373 | 3.62E-06 | D; C | 7.46E-07 | 2.92E-03 | D | 2.57E-01 | D; C | D; C |
| rs113922358 | 6 | 29968681 | 3.62E-06 | D; C | 7.46E-07 | 2.92E-03 | D | 2.57E-01 | D; C | D; C |
| rs3734836 | 6 | 29980073 | 3.62E-06 | D; C | 7.46E-07 | 2.92E-03 | D | 2.57E-01 | D; C | D; C |
| rs60466513 | 6 | 29966609 | 3.62E-06 | D; C | 7.46E-07 | 2.92E-03 | D | 2.57E-01 | D; C | D; C |
| rs6457125 | 6 | 29980570 | 3.62E-06 | D; C | 7.46E-07 | 2.92E-03 | D | 2.57E-01 | D; C | D; C |
| rs6903621 | 6 | 29967486 | 3.62E-06 | D; C | 7.46E-07 | 2.92E-03 | D | 2.57E-01 | D; C | D; C |
| rs6912454 | 6 | 29990044 | 3.62E-06 | D; C | 7.46E-07 | 2.92E-03 | D | 2.57E-01 | D; C | D; C |
| rs6919438 | 6 | 29991649 | 3.62E-06 | D; C | 7.46E-07 | 2.92E-03 | D | 2.57E-01 | D; C | D; C |
| rs6920254 | 6 | 29991798 | 3.62E-06 | D; C | 7.46E-07 | 2.92E-03 | D | 2.57E-01 | D; C | D; C |
| rs6923856 | 6 | 29967529 | 3.62E-06 | D; C | 7.46E-07 | 2.92E-03 | D | 2.57E-01 | D; C | D; C |
| rs6924054 | 6 | 29967618 | 3.62E-06 | D; C | 7.46E-07 | 2.92E-03 | D | 2.57E-01 | D; C | D; C |
| rs6925061 | 6 | 29992286 | 3.62E-06 | D; C | 7.46E-07 | 2.92E-03 | D | 2.57E-01 | D; C | D; C |
| rs9261027 | 6 | 29966000 | 3.62E-06 | D; C | 7.46E-07 | 2.92E-03 | D | 2.57E-01 | D; C | D; C |
| rs9261028 | 6 | 29966017 | 3.62E-06 | D; C | 7.46E-07 | 2.92E-03 | D | 2.57E-01 | D; C | D; C |
| rs9261029 | 6 | 29966041 | 3.62E-06 | D; C | 7.46E-07 | 2.92E-03 | D | 2.57E-01 | D; C | D; C |
| rs9261032 | 6 | 29966124 | 3.62E-06 | D; C | 7.46E-07 | 2.92E-03 | D | 2.57E-01 | D; C | D; C |
| rs9261034 | 6 | 29966141 | 3.62E-06 | D; C | 7.46E-07 | 2.92E-03 | D | 2.57E-01 | D; C | D; C |
| rs9261035 | 6 | 29966175 | 3.62E-06 | D; C | 7.46E-07 | 2.92E-03 | D | 2.57E-01 | D; C | D; C |
| rs9261036 | 6 | 29966301 | 3.62E-06 | D; C | 7.46E-07 | 2.92E-03 | D | 2.57E-01 | D; C | D; C |
| rs9261037 | 6 | 29966465 | 3.62E-06 | D; C | 7.46E-07 | 2.92E-03 | D | 2.57E-01 | D; C | D; C |
| rs9261038 | 6 | 29966513 | 3.62E-06 | D; C | 7.46E-07 | 2.92E-03 | D | 2.57E-01 | D; C | D; C |
| rs9261040 | 6 | 29966620 | 3.62E-06 | D; C | 7.46E-07 | 2.92E-03 | D | 2.57E-01 | D; C | D; C |
| rs9261041 | 6 | 29966718 | 3.62E-06 | D; C | 7.46E-07 | 2.92E-03 | D | 2.57E-01 | D; C | D; C |
| rs9261042 | 6 | 29966721 | 3.62E-06 | D; C | 7.46E-07 | 2.92E-03 | D | 2.57E-01 | D; C | D; C |
| rs9261044 | 6 | 29966749 | 3.62E-06 | D; C | 7.46E-07 | 2.92E-03 | D | 2.57E-01 | D; C | D; C |
| rs9261045 | 6 | 29966867 | 3.62E-06 | D; C | 7.46E-07 | 2.92E-03 | D | 2.57E-01 | D; C | D; C |
| rs9261046 | 6 | 29966998 | 3.62E-06 | D; C | 7.46E-07 | 2.92E-03 | D | 2.57E-01 | D; C | D; C |
| rs9261056 | 6 | 29967799 | 3.62E-06 | D; C | 7.46E-07 | 2.92E-03 | D | 2.57E-01 | D; C | D; C |
| rs9261059 | 6 | 29967999 | 3.62E-06 | D; C | 7.46E-07 | 2.92E-03 | D | 2.57E-01 | D; C | D; C |
| rs9261062 | 6 | 29968122 | 3.62E-06 | D; C | 7.46E-07 | 2.92E-03 | D | 2.57E-01 | D; C | D; C |
| rs9261063 | 6 | 29968306 | 3.62E-06 | D; C | 7.46E-07 | 2.92E-03 | D | 2.57E-01 | D; C | D; C |
| rs9261064 | 6 | 29968374 | 3.62E-06 | D; C | 7.46E-07 | 2.92E-03 | D | 2.57E-01 | D; C | D; C |
| rs9261065 | 6 | 29968394 | 3.62E-06 | D; C | 7.46E-07 | 2.92E-03 | D | 2.57E-01 | D; C | D; C |
| rs9261066 | 6 | 29968407 | 3.62E-06 | D; C | 7.46E-07 | 2.92E-03 | D | 2.57E-01 | D; C | D; C |
| rs9261067 | 6 | 29968457 | 3.62E-06 | D; C | 7.46E-07 | 2.92E-03 | D | 2.57E-01 | D; C | D; C |
| rs9261068 | 6 | 29968458 | 3.62E-06 | D; C | 7.46E-07 | 2.92E-03 | D | 2.57E-01 | D; C | D; C |
| rs9261069 | 6 | 29968499 | 3.62E-06 | D; C | 7.46E-07 | 2.92E-03 | D | 2.57E-01 | D; C | D; C |
| rs9261070 | 6 | 29968507 | 3.62E-06 | D; C | 7.46E-07 | 2.92E-03 | D | 2.57E-01 | D; C | D; C |
| rs9261071 | 6 | 29968557 | 3.62E-06 | D; C | 7.46E-07 | 2.92E-03 | D | 2.57E-01 | D; C | D; C |
| rs9261072 | 6 | 29968564 | 3.62E-06 | D; C | 7.46E-07 | 2.92E-03 | D | 2.57E-01 | D; C | D; C |
| rs9261073 | 6 | 29968578 | 3.62E-06 | D; C | 7.46E-07 | 2.92E-03 | D | 2.57E-01 | D; C | D; C |
| rs9261075 | 6 | 29968621 | 3.62E-06 | D; C | 7.46E-07 | 2.92E-03 | D | 2.57E-01 | D; C | D; C |
| rs9261079 | 6 | 29968764 | 3.62E-06 | D; C | 7.46E-07 | 2.92E-03 | D | 2.57E-01 | D; C | D; C |
| rs9261080 | 6 | 29968987 | 3.62E-06 | D; C | 7.46E-07 | 2.92E-03 | D | 2.57E-01 | D; C | D; C |
| rs9261081 | 6 | 29969040 | 3.62E-06 | D; C | 7.46E-07 | 2.92E-03 | D | 2.57E-01 | D; C | D; C |
| rs9261082 | 6 | 29969199 | 3.62E-06 | D; C | 7.46E-07 | 2.92E-03 | D | 2.57E-01 | D; C | D; C |
| rs9261083 | 6 | 29969205 | 3.62E-06 | D; C | 7.46E-07 | 2.92E-03 | D | 2.57E-01 | D; C | D; C |
| rs9261084 | 6 | 29969223 | 3.62E-06 | D; C | 7.46E-07 | 2.92E-03 | D | 2.57E-01 | D; C | D; C |
| rs9261085 | 6 | 29969513 | 3.62E-06 | D; C | 7.46E-07 | 2.92E-03 | D | 2.57E-01 | D; C | D; C |
| rs9261086 | 6 | 29969596 | 3.62E-06 | D; C | 7.46E-07 | 2.92E-03 | D | 2.57E-01 | D; C | D; C |
| rs9261087 | 6 | 29969602 | 3.62E-06 | D; C | 7.46E-07 | 2.92E-03 | D | 2.57E-01 | D; C | D; C |
| rs9261088 | 6 | 29969646 | 3.62E-06 | D; C | 7.46E-07 | 2.92E-03 | D | 2.57E-01 | D; C | D; C |
| rs9261089 | 6 | 29969818 | 3.62E-06 | D; C | 7.46E-07 | 2.92E-03 | D | 2.57E-01 | D; C | D; C |
| rs9261090 | 6 | 29969929 | 3.62E-06 | D; C | 7.46E-07 | 2.92E-03 | D | 2.57E-01 | D; C | D; C |
| rs9261091 | 6 | 29969973 | 3.62E-06 | D; C | 7.46E-07 | 2.92E-03 | D | 2.57E-01 | D; C | D; C |
| rs9261093 | 6 | 29970685 | 3.62E-06 | D; C | 7.46E-07 | 2.92E-03 | D | 2.57E-01 | D; C | D; C |
| rs9261094 | 6 | 29970839 | 3.62E-06 | D; C | 7.46E-07 | 2.92E-03 | D | 2.57E-01 | D; C | D; C |
| rs9261130 | 6 | 29980445 | 3.62E-06 | D; C | 7.46E-07 | 2.92E-03 | D | 2.57E-01 | D; C | D; C |
| rs9261133 | 6 | 29981187 | 3.62E-06 | D; C | 7.46E-07 | 2.92E-03 | D | 2.57E-01 | D; C | D; C |
| rs9261134 | 6 | 29981368 | 3.62E-06 | D; C | 7.46E-07 | 2.92E-03 | D | 2.57E-01 | D; C | D; C |
| rs9261135 | 6 | 29981378 | 3.62E-06 | D; C | 7.46E-07 | 2.92E-03 | D | 2.57E-01 | D; C | D; C |
| rs9261138 | 6 | 29981515 | 3.62E-06 | D; C | 7.46E-07 | 2.92E-03 | D | 2.57E-01 | D; C | D; C |
| rs9261141 | 6 | 29983327 | 3.62E-06 | D; C | 7.46E-07 | 2.92E-03 | D | 2.57E-01 | D; C | D; C |
| rs9261146 | 6 | 29985325 | 3.62E-06 | D; C | 7.46E-07 | 2.92E-03 | D | 2.57E-01 | D; C | D; C |
| rs9261147 | 6 | 29985488 | 3.62E-06 | D; C | 7.46E-07 | 2.92E-03 | D | 2.57E-01 | D; C | D; C |
| rs9261156 | 6 | 29991538 | 3.62E-06 | D; C | 7.46E-07 | 2.92E-03 | D | 2.57E-01 | D; C | D; C |
| rs9261158 | 6 | 29992953 | 3.62E-06 | D; C | 7.46E-07 | 2.92E-03 | D | 2.57E-01 | D; C | D; C |
| rs9261159 | 6 | 29992973 | 3.62E-06 | D; C | 7.46E-07 | 2.92E-03 | D | 2.57E-01 | D; C | D; C |
| rs9261169 | 6 | 29995664 | 3.62E-06 | D; C | 7.46E-07 | 2.92E-03 | D | 2.57E-01 | D; C | D; C |
| rs9261171 | 6 | 29996121 | 3.62E-06 | D; C | 7.46E-07 | 2.92E-03 | D | 2.57E-01 | D; C | D; C |
| rs9261175 | 6 | 29996986 | 3.62E-06 | D; C | 7.46E-07 | 2.92E-03 | D | 2.57E-01 | D; C | D; C |
| rs9261176 | 6 | 29997312 | 3.62E-06 | D; C | 7.46E-07 | 2.92E-03 | D | 2.57E-01 | D; C | D; C |
| rs9261177 | 6 | 29997583 | 3.62E-06 | D; C | 7.46E-07 | 2.92E-03 | D | 2.57E-01 | D; C | D; C |
| rs9261179 | 6 | 29998260 | 3.62E-06 | D; C | 7.46E-07 | 2.92E-03 | D | 2.57E-01 | D; C | D; C |
| rs9261184 | 6 | 29999185 | 3.62E-06 | D; C | 7.46E-07 | 2.92E-03 | D | 2.57E-01 | D; C | D; C |
| rs9280837 | 6 | 29969330 | 3.62E-06 | D; C | 7.46E-07 | 2.92E-03 | D | 2.57E-01 | D; C | D; C |
| rs9280850 | 6 | 29994294 | 3.62E-06 | D; C | 7.46E-07 | 2.92E-03 | D | 2.57E-01 | D; C | D; C |
| rs9261360 | 6 | 30055667 | 3.64E-06 | D; C | 6.90E-07 | 3.22E-03 | D | 2.63E-01 | D; C | D; C |
| rs375934146 | 6 | 30043971 | 3.67E-06 | D; C | 6.94E-07 | 2.95E-03 | D | 2.45E-01 | D; C | D; C |
| rs3807036 | 6 | 30044914 | 3.67E-06 | D; C | 6.94E-07 | 2.95E-03 | D | 2.45E-01 | D; C | D; C |
| rs9261312 | 6 | 30046540 | 3.67E-06 | D; C | 6.94E-07 | 2.95E-03 | D | 2.45E-01 | D; C | D; C |
| rs17337582 | 5 | 57040318 | 3.69E-06 | C; M | 4.49E-06 | 1.38E-02 | C | 1.18E-01 | C; M | C; M |
| rs62358383 | 5 | 57040466 | 3.69E-06 | C; M | 4.49E-06 | 1.38E-02 | C | 1.18E-01 | C; M | C; M |
| rs67675244 | 4 | 181524731 | 3.75E-06 | M | 8.59E-05 | 1.67E-01 | M | - | - | M |
| rs3893185 | 4 | 6874834 | 3.76E-06 | M | 5.29E-05 | 8.43E-02 | M | - | - | M |
| rs2539731 | 5 | 57073535 | 3.84E-06 | C; M | 1.55E-06 | 2.27E-03 | C | 7.40E-02 | C; M | C; M |
| rs3891156 | 6 | 29988740 | 3.92E-06 | D; C | 8.07E-07 | 3.04E-03 | D | 2.50E-01 | D; C | D; C |
| rs3891157 | 6 | 29988439 | 3.92E-06 | D; C | 8.07E-07 | 3.04E-03 | D | 2.50E-01 | D; C | D; C |
| rs9261151 | 6 | 29987738 | 3.92E-06 | D; C | 8.07E-07 | 3.04E-03 | D | 2.50E-01 | D; C | D; C |
| rs12215270 | 6 | 133157237 | 3.94E-06 | M | 1.04E-04 | 2.72E-01 | M | - | - | M |
| rs142996705 | 6 | 133157241 | 3.94E-06 | M | 1.04E-04 | 2.72E-01 | M | - | - | M |
| rs10998317 | 10 | 70358197 | 4.03E-06 | C | 3.51E-04 | 6.56E-02 | C | - | - | C |
| rs2664425 | 10 | 70371089 | 4.25E-06 | C; M | 1.12E-04 | 1.47E-03 | C | 7.74E-02 | C; M | C; M |
| rs142611169 | 10 | 120561628 | 4.37E-06 | D; C | 5.43E-06 | 1.56E-03 | C | 7.62E-01 | D; C | D; C |
| rs34411702 | 4 | 6835635 | 4.38E-06 | M | 7.90E-05 | 1.73E-01 | M | - | - | M |
| rs2664419 | 10 | 70378139 | 4.44E-06 | C; M | 1.01E-04 | 1.20E-03 | D | 5.45E-02 | C; M | C; M |
| rs9415926 | 10 | 70376994 | 4.44E-06 | C; M | 1.01E-04 | 1.20E-03 | D | 5.45E-02 | C; M | C; M |
| rs1019478 | 4 | 181525745 | 4.68E-06 | D; M | 3.09E-05 | 1.06E-02 | M | 8.56E-01 | D; M | D; M |
| rs111921679 | 5 | 78088178 | 4.72E-06 | M | 1.93E-06 | 5.36E-01 | M | - | - | M |
| rs112979037 | 5 | 78088119 | 4.72E-06 | M | 1.93E-06 | 5.36E-01 | M | - | - | M |
| rs6924273 | 6 | 29967810 | 4.72E-06 | D; C | 1.52E-06 | 3.79E-03 | D | 2.94E-01 | D; C | D; C |
| rs370285 | 5 | 67676904 | 4.75E-06 | D; C; M | 2.51E-07 | 4.74E-05 | C | 2.09E-02 | D; C | D; C; M |
| rs3910652 | 5 | 78087584 | 4.80E-06 | M | 1.67E-06 | 5.23E-01 | M | - | - | M |
| rs7249558 | 19 | 53789891 | 4.83E-06 | C; M | 3.56E-06 | 1.51E-02 | C | 1.21E-01 | C; M | C; M |
| rs11118425 | 1 | 207879626 | 4.87E-06 | M | 1.80E-02 | 3.16E-01 | M | - | - | M |
| rs10998310 | 10 | 70348685 | 4.89E-06 | C; M | 4.09E-04 | 3.86E-02 | C | 6.05E-01 | C; M | C; M |
| rs141782667 | 10 | 91219273 | 4.90E-06 | M | 4.45E-08 | 8.90E-01 | M | - | - | M |
| rs1542562 | 12 | 25738937 | 4.91E-06 | C | 8.81E-06 | 6.18E-02 | C | - | - | C |
| rs1543933 | 12 | 25739159 | 4.91E-06 | C | 8.81E-06 | 6.18E-02 | C | - | - | C |
| rs10823237 | 10 | 70388920 | 5.16E-06 | C; M | 9.01E-05 | 1.13E-03 | C | 5.47E-02 | C; M | C; M |
| rs34622339 | 10 | 70393125 | 5.16E-06 | C; M | 9.01E-05 | 1.13E-03 | C | 5.47E-02 | C; M | C; M |
| rs6814754 | 4 | 6860740 | 5.16E-06 | M | 6.34E-05 | 1.21E-01 | M | - | - | M |
| rs145679846 | 6 | 30024686 | 5.18E-06 | D; C | 1.38E-06 | 4.00E-03 | D | 2.91E-01 | D; C | D; C |
| rs10998342 | 10 | 70386834 | 5.20E-06 | C; M | 9.33E-05 | 1.13E-03 | C | 5.52E-02 | C; M | C; M |
| rs73276500 | 10 | 70365455 | 5.21E-06 | C | 4.02E-04 | 5.67E-02 | C | - | - | C |
| rs77560985 | 10 | 70363263 | 5.30E-06 | C | 3.78E-04 | 6.03E-02 | C | - | - | C |
| rs6833851 | 4 | 6854625 | 5.41E-06 | M | 3.96E-05 | 2.54E-01 | M | - | - | M |
| rs3091238 | 19 | 53783181 | 5.41E-06 | C | 1.21E-05 | 9.77E-02 | C | - | - | C |
| rs3113752 | 4 | 181519492 | 5.42E-06 | M | 1.24E-05 | 2.90E-01 | M | - | - | M |
| rs34319433 | 4 | 181522625 | 5.58E-06 | M | 4.63E-05 | 7.99E-02 | M | - | - | M |
| rs7659806 | 4 | 181522815 | 5.58E-06 | M | 4.63E-05 | 7.99E-02 | M | - | - | M |
| rs7682048 | 4 | 181522829 | 5.58E-06 | M | 4.63E-05 | 7.99E-02 | M | - | - | M |
| rs994347 | 4 | 181522926 | 5.58E-06 | M | 4.63E-05 | 7.99E-02 | M | - | - | M |
| rs1895263 | 4 | 181523283 | 5.71E-06 | M | 4.68E-05 | 6.62E-02 | M | - | - | M |
| rs994345 | 4 | 181523287 | 5.71E-06 | M | 4.68E-05 | 6.62E-02 | M | - | - | M |
| rs142082144 | 10 | 70343922 | 5.75E-06 | C; M | 6.93E-04 | 4.85E-02 | C | 6.61E-01 | C; M | C; M |
| rs12662842 | 6 | 170782518 | 5.80E-06 | D; M | 7.41E-07 | 1.20E-03 | M | 7.58E-01 | D; M | D; M |
| rs73028371 | 6 | 170782670 | 5.80E-06 | D; M | 7.41E-07 | 1.20E-03 | M | 7.58E-01 | D; M | D; M |
| rs9460196 | 6 | 170783347 | 5.80E-06 | D; M | 7.41E-07 | 1.20E-03 | M | 7.58E-01 | D; M | D; M |
| rs9460197 | 6 | 170783832 | 5.80E-06 | D; M | 7.41E-07 | 1.20E-03 | M | 7.58E-01 | D; M | D; M |
| rs9460198 | 6 | 170786827 | 5.80E-06 | D; M | 7.41E-07 | 1.20E-03 | M | 7.58E-01 | D; M | D; M |
| rs10967764 | 9 | 27189869 | 5.84E-06 | D; C; M | 4.71E-08 | 6.19E-04 | M | 3.84E-02 | C; M | D; C; M |
| rs78940997 | 7 | 53019158 | 5.98E-06 | C | 9.94E-06 | 1.53E-01 | C | - | - | C |
| rs4570681 | 12 | 25729001 | 5.99E-06 | C | 1.16E-05 | 7.51E-02 | C | - | - | C |
| rs35824020 | 4 | 14271701 | 6.00E-06 | C | 4.23E-06 | 2.71E-01 | C | - | - | C |
| rs9548658 | 13 | 31508890 | 6.07E-06 | D; C; M | 2.75E-08 | 1.80E-06 | C | 4.54E-02 | C; M | D; C; M |
| rs4699351 | 4 | 99496018 | 6.30E-06 | C; M | 2.41E-05 | 5.84E-03 | C | 6.63E-02 | C; M | C; M |
| rs141594731 | 12 | 116725560 | 6.43E-06 | C | 2.75E-06 | 2.66E-01 | C | - | - | C |
| rs994346 | 4 | 181523057 | 6.46E-06 | M | 6.31E-05 | 7.98E-02 | M | - | - | M |
| rs4823965 | 22 | 49394161 | 6.71E-06 | D; C | 5.82E-06 | 1.96E-02 | D | 7.13E-01 | D; C | D; C |
| rs12498491 | 4 | 179335309 | 6.73E-06 | M | 2.64E-03 | 5.17E-02 | M | - | - | M |
| rs140493770 | 21 | 43148689 | 6.80E-06 | D; M | 7.51E-06 | 3.61E-03 | M | 5.03E-01 | D; M | D; M |
| rs7673297 | 4 | 181524906 | 6.80E-06 | D; M | 3.29E-05 | 1.29E-02 | M | 7.73E-01 | D; M | D; M |
| rs67201270 | 10 | 70351054 | 7.06E-06 | C; M | 7.70E-04 | 4.74E-02 | C | 6.54E-01 | C; M | C; M |
| rs7395060 | 11 | 119703617 | 7.07E-06 | D; C; M | 2.17E-06 | 1.41E-03 | D | 3.48E-02 | D; M | D; C; M |
| rs62366622 | 5 | 58051937 | 7.09E-06 | D; C | 2.17E-04 | 2.25E-02 | C | 1.30E-01 | D; C | D; C |
| rs4312758 | 4 | 6874517 | 7.13E-06 | M | 9.81E-05 | 1.79E-01 | M | - | - | M |
| rs6845985 | 4 | 6855767 | 7.16E-06 | M | 5.42E-05 | 1.17E-01 | M | - | - | M |
| rs7912984 | 10 | 70343285 | 7.21E-06 | C; M | 4.71E-04 | 3.99E-02 | C | 6.78E-01 | C; M | C; M |
| rs2865243 | 19 | 53787841 | 7.34E-06 | C | 1.81E-05 | 9.66E-02 | C | - | - | C |
| rs79154919 | 4 | 14732089 | 7.41E-06 | M | 2.10E-06 | 2.72E-01 | M | - | - | M |
| rs13208577 | 6 | 24602347 | 7.67E-06 | C | 1.43E-06 | 7.63E-01 | C | - | - | C |
| rs12854887 | 23 | 51338769 | 7.80E-06 | D | 3.76E-06 | 3.42E-01 | D | - | - | D |
| rs188021672 | 5 | 116024504 | 7.94E-06 | C | 3.37E-04 | 1.61E-01 | C | - | - | C |
| rs1858608 | 10 | 95970288 | 7.96E-06 | C | 2.16E-06 | 3.93E-01 | C | - | - | C |
| rs75337932 | 4 | 188968298 | 8.00E-06 | M | 8.51E-05 | 7.18E-02 | M | - | - | M |
| rs13103065 | 4 | 6815311 | 8.01E-06 | M | 4.90E-05 | 8.52E-01 | M | - | - | M |
| rs530061637 | 1 | 27061626 | 8.02E-06 | C | 1.37E-07 | 6.06E-01 | C | - | - | C |
| rs12843829 | 23 | 51240725 | 8.07E-06 | D | 6.03E-06 | 2.68E-01 | D | - | - | D |
| rs72833931 | 2 | 69689509 | 8.19E-06 | C; M | 2.84E-08 | 7.77E-04 | M | 3.11E-01 | C; M | C; M |
| rs10823229 | 10 | 70332580 | 8.21E-06 | C; M | 6.93E-04 | 4.85E-02 | C | 6.61E-01 | C; M | C; M |
| rs10823231 | 10 | 70338978 | 8.21E-06 | C; M | 6.93E-04 | 4.85E-02 | C | 6.61E-01 | C; M | C; M |
| rs10998295 | 10 | 70336060 | 8.21E-06 | C; M | 6.93E-04 | 4.85E-02 | C | 6.61E-01 | C; M | C; M |
| rs10998298 | 10 | 70338538 | 8.21E-06 | C; M | 6.93E-04 | 4.85E-02 | C | 6.61E-01 | C; M | C; M |
| rs10998299 | 10 | 70338575 | 8.21E-06 | C; M | 6.93E-04 | 4.85E-02 | C | 6.61E-01 | C; M | C; M |
| rs111911118 | 10 | 70338218 | 8.21E-06 | C; M | 6.93E-04 | 4.85E-02 | C | 6.61E-01 | C; M | C; M |
| rs12241884 | 10 | 70340396 | 8.21E-06 | C; M | 6.93E-04 | 4.85E-02 | C | 6.61E-01 | C; M | C; M |
| rs144742224 | 10 | 70353189 | 8.21E-06 | C; M | 6.93E-04 | 4.85E-02 | C | 6.61E-01 | C; M | C; M |
| rs57523358 | 10 | 70334944 | 8.21E-06 | C; M | 6.93E-04 | 4.85E-02 | C | 6.61E-01 | C; M | C; M |
| rs58968626 | 10 | 70335082 | 8.21E-06 | C; M | 6.93E-04 | 4.85E-02 | C | 6.61E-01 | C; M | C; M |
| rs60687524 | 10 | 70338462 | 8.21E-06 | C; M | 6.93E-04 | 4.85E-02 | C | 6.61E-01 | C; M | C; M |
| rs67117966 | 10 | 70336333 | 8.21E-06 | C; M | 6.93E-04 | 4.85E-02 | C | 6.61E-01 | C; M | C; M |
| rs68122210 | 10 | 70336270 | 8.21E-06 | C; M | 6.93E-04 | 4.85E-02 | C | 6.61E-01 | C; M | C; M |
| rs7896907 | 10 | 70343537 | 8.21E-06 | C; M | 6.93E-04 | 4.85E-02 | C | 6.61E-01 | C; M | C; M |
| rs7908570 | 10 | 70348972 | 8.21E-06 | C; M | 6.93E-04 | 4.85E-02 | C | 6.61E-01 | C; M | C; M |
| rs12243361 | 10 | 70351520 | 8.26E-06 | C; M | 8.36E-04 | 4.65E-02 | C | 6.62E-01 | C; M | C; M |
| rs72637095 | 10 | 70352034 | 8.26E-06 | C; M | 8.36E-04 | 4.65E-02 | C | 6.62E-01 | C; M | C; M |
| rs72797581 | 10 | 70353313 | 8.26E-06 | C; M | 8.36E-04 | 4.65E-02 | C | 6.62E-01 | C; M | C; M |
| rs78360461 | 10 | 70352370 | 8.26E-06 | C; M | 8.36E-04 | 4.65E-02 | C | 6.62E-01 | C; M | C; M |
| rs112134609 | 4 | 7093203 | 8.45E-06 | C; M | 1.09E-06 | 1.70E-02 | M | 5.77E-02 | C; M | C; M |
| rs56110003 | 4 | 7092161 | 8.45E-06 | C; M | 1.09E-06 | 1.70E-02 | M | 5.77E-02 | C; M | C; M |
| rs3960965 | 19 | 53789383 | 8.48E-06 | C | 4.15E-05 | 5.24E-02 | C | - | - | C |
| rs35064938 | 23 | 51331771 | 8.51E-06 | D | 6.64E-06 | 2.95E-01 | D | - | - | D |
| rs111312303 | 23 | 51235423 | 8.73E-06 | D | 6.33E-06 | 2.64E-01 | D | - | - | D |
| rs113220712 | 23 | 51287134 | 8.73E-06 | D | 6.33E-06 | 2.64E-01 | D | - | - | D |
| rs12832410 | 23 | 51230625 | 8.73E-06 | D | 6.33E-06 | 2.64E-01 | D | - | - | D |
| rs12833954 | 23 | 51282161 | 8.73E-06 | D | 6.33E-06 | 2.64E-01 | D | - | - | D |
| rs12840468 | 23 | 51282586 | 8.73E-06 | D | 6.33E-06 | 2.64E-01 | D | - | - | D |
| rs12842085 | 23 | 51269539 | 8.73E-06 | D | 6.33E-06 | 2.64E-01 | D | - | - | D |
| rs12842762 | 23 | 51279476 | 8.73E-06 | D | 6.33E-06 | 2.64E-01 | D | - | - | D |
| rs12844177 | 23 | 51254920 | 8.73E-06 | D | 6.33E-06 | 2.64E-01 | D | - | - | D |
| rs12845472 | 23 | 51209675 | 8.73E-06 | D | 6.33E-06 | 2.64E-01 | D | - | - | D |
| rs12849901 | 23 | 51283932 | 8.73E-06 | D | 6.33E-06 | 2.64E-01 | D | - | - | D |
| rs12854262 | 23 | 51237340 | 8.73E-06 | D | 6.33E-06 | 2.64E-01 | D | - | - | D |
| rs12860343 | 23 | 51286478 | 8.73E-06 | D | 6.33E-06 | 2.64E-01 | D | - | - | D |
| rs139832819 | 23 | 51260081 | 8.73E-06 | D | 6.33E-06 | 2.64E-01 | D | - | - | D |
| rs151219484 | 23 | 51256761 | 8.73E-06 | D | 6.33E-06 | 2.64E-01 | D | - | - | D |
| rs78269975 | 23 | 51275624 | 8.73E-06 | D | 6.33E-06 | 2.64E-01 | D | - | - | D |
| rs2865242 | 19 | 53787832 | 8.75E-06 | C; M | 1.69E-05 | 4.03E-02 | C | 2.77E-01 | C; M | C; M |
| rs2488047 | 10 | 70371743 | 8.76E-06 | C; M | 2.12E-04 | 2.08E-03 | D | 6.00E-02 | C; M | C; M |
| rs3975351 | 19 | 53789549 | 8.90E-06 | C; M | 2.24E-05 | 3.40E-02 | C | 2.97E-01 | C; M | C; M |
| rs71727226 | 13 | 31498152 | 8.98E-06 | D; C; M | 5.33E-08 | 3.60E-06 | C | 4.83E-02 | C; M | D; C; M |
| rs12479371 | 2 | 139465016 | 9.08E-06 | D; M | 2.39E-05 | 5.15E-03 | M | 1.05E-01 | D; M | D; M |
| rs376782312 | 10 | 70371431 | 9.23E-06 | C; M | 3.13E-04 | 2.29E-03 | D | 7.02E-02 | C; M | C; M |
| rs12648289 | 4 | 181525582 | 9.24E-06 | D; M | 2.01E-05 | 1.72E-02 | M | 8.14E-01 | D; M | D; M |
| rs13123843 | 4 | 181525569 | 9.24E-06 | D; M | 2.01E-05 | 1.72E-02 | M | 8.14E-01 | D; M | D; M |
| rs1187604695 | 4 | 7094075 | 9.29E-06 | C; M | 1.13E-06 | 1.68E-02 | M | 5.54E-02 | C; M | C; M |
| rs12850240 | 23 | 51292502 | 9.30E-06 | D | 1.36E-05 | 3.33E-01 | D | - | - | D |
| rs11497984 | 10 | 70337170 | 9.36E-06 | C; M | 6.93E-04 | 4.85E-02 | C | 6.61E-01 | C; M | C; M |
| rs7894063 | 10 | 70349116 | 9.36E-06 | C; M | 6.93E-04 | 4.85E-02 | C | 6.61E-01 | C; M | C; M |
| rs74577155 | 10 | 120561308 | 9.40E-06 | D; C | 9.16E-06 | 2.31E-03 | C | 9.21E-01 | D; C | D; C |
| rs12371702 | 12 | 13855377 | 9.82E-06 | D; M | 4.92E-06 | 7.69E-03 | M | 8.71E-02 | D; M | D; M |
| rs10998287 | 10 | 70330892 | 9.82E-06 | C | 8.42E-04 | 5.48E-02 | C | - | - | C |
| rs10998288 | 10 | 70331038 | 9.82E-06 | C | 8.42E-04 | 5.48E-02 | C | - | - | C |

SNP, nucleotide polymorphism; Chr, chromosome; BP, base pair; D, depression; C, cognition; M, memory.

^a^ The *P* value was derived from multivariate GWAS.

^b^ Sequential tests of pleiotropy with a *P* threshold of 0.05.

^c^ Single test of the number of phenotypes associated with genotype, H0 (test 0): all betas = 0.

^d^ Single test of the number of phenotypes associated with genotype, H0 (test 1): one or less beta is nonzero.

^e^ Single test of the number of phenotypes associated with genotype, H0 (test 2): two or less betas are nonzero.
