# Supplementary material for: Multivariate genome-wide association study of depression, cognition, and memory phenotypes and validation analysis identify 12 cross-ethnic variants
Source: Transl Psychiatry. 2022 Jul 30;12:304. doi: 10.1038/s41398-022-02074-x (PMC9338946; doi:10.1038/s41398-022-02074-x)
Supplement: Supplementary file 5 — Supplementary Table 5 [file 41398_2022_2074_MOESM5_ESM.doc]

**Supplementary Table 5** The top 20 SNPs from multivariate GWAS of depression-cognition-memory after imputation

| SNP | Chr | Band | BP | *P* value | Gene or nearest gene | Official full name |
| --- | --- | --- | --- | --- | --- | --- |
| chr6:24597173 | 6 | 6p22.3 | 24597173 | 1.71E-07 | *KIAA0319* | KIAA0319 |
| rs12210323 | 6 | 6p22.3 | 24591728 | 1.71E-07 | *KIAA0319* | KIAA0319 |
| rs12213116 | 6 | 6p22.3 | 24594630 | 1.72E-07 | *KIAA0319* | KIAA0319 |
| rs61783213 | 1 | 1p31.1 | 77260333 | 2.77E-07 | *LINC02567* | long intergenic non-protein coding RNA 2567 |
| rs9589468 | 13 | 13q31.3 | 92916268 | 3.25E-07 | *GPC5* | glypican 5 |
| rs67007022 | 4 | 4q34.3 | 181525540 | 3.46E-07 | *NDUFB5P1* | NADH:ubiquinone oxidoreductase subunit B5 pseudogene 1 |
| rs533752605 | 3 | 3p12.1 | 84415473 | 3.55E-07 | *LINC00971* | long intergenic non-protein coding RNA 971 |
| rs11577464 | 1 | 1p31.1 | 77259107 | 5.72E-07 | *LINC02567* | long intergenic non-protein coding RNA 2567 |
| rs112897238 | 1 | 1p31.1 | 77260102 | 6.31E-07 | *LINC02567* | long intergenic non-protein coding RNA 2567 |
| rs73198369 | 4 | 4q13.1 | 60865968 | 6.93E-07 | *RNU6-1325P* | RNA, U6 small nuclear 1325, pseudogene |
| rs374850664 | 10 | 10q21.3 | 70336105 | 7.36E-07 | *TET1* | tet methylcytosine dioxygenase 1 |
| rs8036389 | 15 | 15q26.2 | 97492430 | 7.46E-07 | *RN7SKP181* | RN7SK pseudogene 181 |
| rs11599886 | 10 | 10q21.3 | 70324991 | 8.42E-07 | *TET1* | tet methylcytosine dioxygenase 1 |
| rs7898805 | 10 | 10q26.11 | 120566433 | 8.55E-07 | *CACUL1* | CDK2 associated cullin domain 1 |
| rs58350164 | 10 | 10q26.11 | 120568419 | 8.99E-07 | *CACUL1* | CDK2 associated cullin domain 1 |
| rs61012528 | 10 | 10q26.11 | 120568716 | 9.79E-07 | *CACUL1* | CDK2 associated cullin domain 1 |
| rs9589470 | 13 | 13q31.3 | 92917856 | 1.18E-06 | *GPC5* | glypican 5 |
| rs117254295 | 10 | 10q26.11 | 120563039 | 1.30E-06 | *CACUL1* | CDK2 associated cullin domain 1 |
| rs34631623 | 18 | 18q12.1 | 29438021 | 1.38E-06 | *TRAPPC8* | trafficking protein particle complex 8 |
| rs79882698 | 10 | 10q26.11 | 120569407 | 1.41E-06 | *CACUL1* | CDK2 associated cullin domain 1 |

SNP, nucleotide polymorphism; Chr, chromosome; BP, base pair.
