# Supplementary material for: Multivariate genome-wide association study of depression, cognition, and memory phenotypes and validation analysis identify 12 cross-ethnic variants
Source: Transl Psychiatry. 2022 Jul 30;12:304. doi: 10.1038/s41398-022-02074-x (PMC9338946; doi:10.1038/s41398-022-02074-x)
Supplement: Supplementary file 6 — Supplementary Table 6 [file 41398_2022_2074_MOESM6_ESM.doc]

**Supplementary Table 6** The SNPs with nominal significance (*P*<0.05) in UK Biobank validation analysis

| SNP | Chr | Band | BP | OR | *P* value | Located gene | eQTL gene (in brain tissue) |
| --- | --- | --- | --- | --- | --- | --- | --- |
| rs2539731 | 5 | 5q11.2 | 57073535 | 1.37 | 5.88E-04 | *-* | *MAP3K1* |
| rs17337582 | 5 | 5q11.2 | 57040318 | 1.32 | 2.30E-03 | *-* | *MAP3K1* |
| rs62358383 | 5 | 5q11.2 | 57040466 | 1.32 | 2.52E-03 | *-* | *MAP3K1* |
| rs2041433 | 15 | 15q26.2 | 97493539 | 1.29 | 6.52E-03 | *-* | *-* |
| rs7250500 | 19 | 19q13.4 | 53789853 | 0.75 | 1.67E-02 | *-* | *-* |
| rs8036389 | 15 | 15q26.2 | 97492430 | 1.25 | 2.11E-02 | *-* | *-* |
| rs2865242 | 19 | 19q13.4 | 53787832 | 0.77 | 2.32E-02 | *FAM90A27P* | *-* |
| rs13209442 | 6 | 6p22.3 | 24597176 | 1.47 | 4.33E-02 | *KIAA0319* | *-* |
| rs9261134 | 6 | 6p22.1 | 29981368 | 0.65 | 4.34E-02 | *ZNRD1ASP* | *HCG4, HCG4P3, HCP5B, HLA-A, HLA-F, HLA-F-AS1, HLA-H, HLA-K, HLA-V, HLA-W, PPP1R11, RNF39, RPL23AP1,* *ZNRD1, ZNRD1ASP* |
| rs13208577 | 6 | 6p22.3 | 24602347 | 1.46 | 4.60E-02 | *KIAA0319* | *-* |
| rs12213116 | 6 | 6p22.3 | 24594630 | 1.46 | 4.63E-02 | *KIAA0319* | *-* |
| rs4312758 | 4 | 4p16.1 | 6874517 | 0.75 | 4.70E-02 | *KIAA0232* | *-* |

SNP, nucleotide polymorphism; Chr, chromosome; BP, base pair; eQTL, expression quantitative trait loci.
