# Supplementary material for: Multivariate genome-wide association study of depression, cognition, and memory phenotypes and validation analysis identify 12 cross-ethnic variants
Source: Transl Psychiatry. 2022 Jul 30;12:304. doi: 10.1038/s41398-022-02074-x (PMC9338946; doi:10.1038/s41398-022-02074-x)
Supplement: Supplementary file 7 — Supplementary figure and table legends [file 41398_2022_2074_MOESM7_ESM.docx]

**Supplementary figure and table legends**

**Supplementary Figure 1** Bulk tissue gene expression for *KIAA0319* from the GTEx database. The horizontal axis represents different types of tissues, while the vertical axis represents the expression values. TPM, Transcripts per million.

**Supplementary Figure 2** Expression quantitative trait loci (eQTL) analysis of rs2539731 with *MAP3K1* across tissue types from the GTEx database. NES is the normalized effect size (*β*) from single-tissue eQTL analysis. The *P* value is from the *t*-test that compares observed NES in single-tissue eQTL analysis to the null hypothesis of no NES. The m-value represents a posterior probability that the effect of eQTL exists in each tissue of a cross-tissue meta-analysis.

**Supplementary Figure 3** Expression quantitative trait loci (eQTL) analysis of rs17337582 with *MAP3K1* across tissue types from the GTEx database. NES is the normalized effect size (*β*) from single-tissue eQTL analysis. The *P* value is from the *t*-test that compares observed NES in single-tissue eQTL analysis to the null hypothesis of no NES. The m-value represents a posterior probability that the effect of eQTL exists in each tissue of a cross-tissue meta-analysis.

**Supplementary Figure 4** Expression quantitative trait loci (eQTL) analysis of rs62358383 with *MAP3K1* across tissue types from the GTEx database. NES is the normalized effect size (*β*) from single-tissue eQTL analysis. The *P* value is from the *t*-test that compares observed NES in single-tissue eQTL analysis to the null hypothesis of no NES. The m-value represents a posterior probability that the effect of eQTL exists in each tissue of a cross-tissue meta-analysis.

**Supplementary Figure 5** Expression quantitative trait loci (eQTL) analysis of rs9261134 with *ZNRD1ASP* across tissue types from the GTEx database. NES is the normalized effect size (*β*) from single-tissue eQTL analysis. The *P* value is from the *t*-test that compares observed NES in single-tissue eQTL analysis to the null hypothesis of no NES. The m-value represents a posterior probability that the effect of eQTL exists in each tissue of a cross-tissue meta-analysis.

**Supplementary Table 1** Basic characteristics of participants in the discovery phase.

**Supplementary Table 2** The results of pleiotropy analysis for multivariate GWAS of depression-cognition-memory identified SNPs (*P*<1×10^-5^).

**Supplementary Table 3** The enhancer enrichment results of top 100 depression-cognition-memory-related SNPs.

**Supplementary Table 4** The results of pleiotropy analysis for multivariate GWAS of depression-cognition-memory identified SNPs (*P*<1×10^-5^) after imputation.

**Supplementary Table 5** The top 20 SNPs from multivariate GWAS of depression-cognition-memory after imputation.

**Supplementary Table 6** The SNPs with nominal significance (*P*<0.05) in UK Biobank validation analysis.
