# Supplementary figures and images for: Multivariate genome-wide association study of depression, cognition, and memory phenotypes and validation analysis identify 12 cross-ethnic variants
Source: Transl Psychiatry. 2022 Jul 30;12:304. doi: 10.1038/s41398-022-02074-x (PMC9338946; doi:10.1038/s41398-022-02074-x)

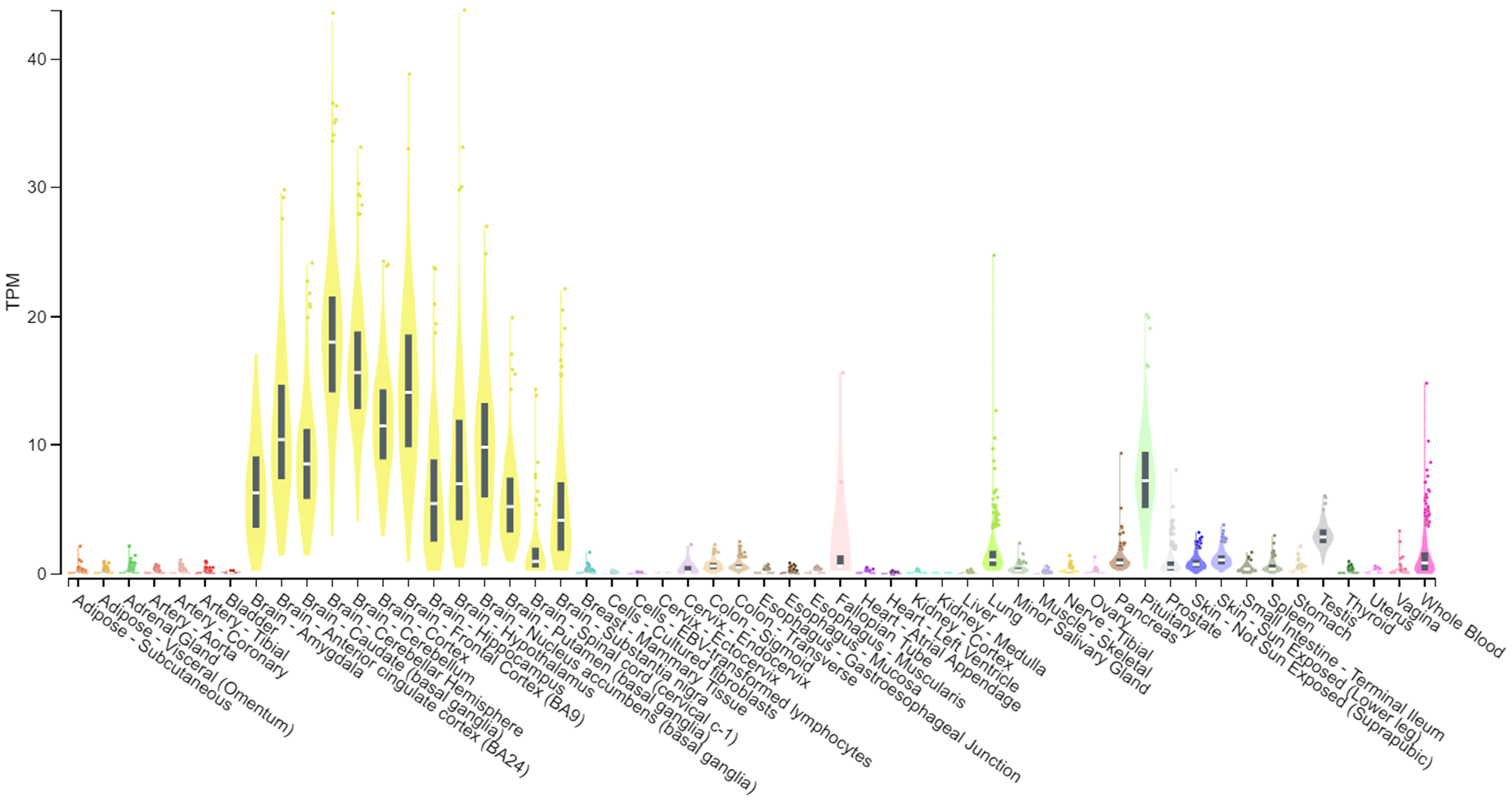

Supplement: Supplementary file 8 — Supplementary Figure 1 [file 41398_2022_2074_MOESM8_ESM.tif]

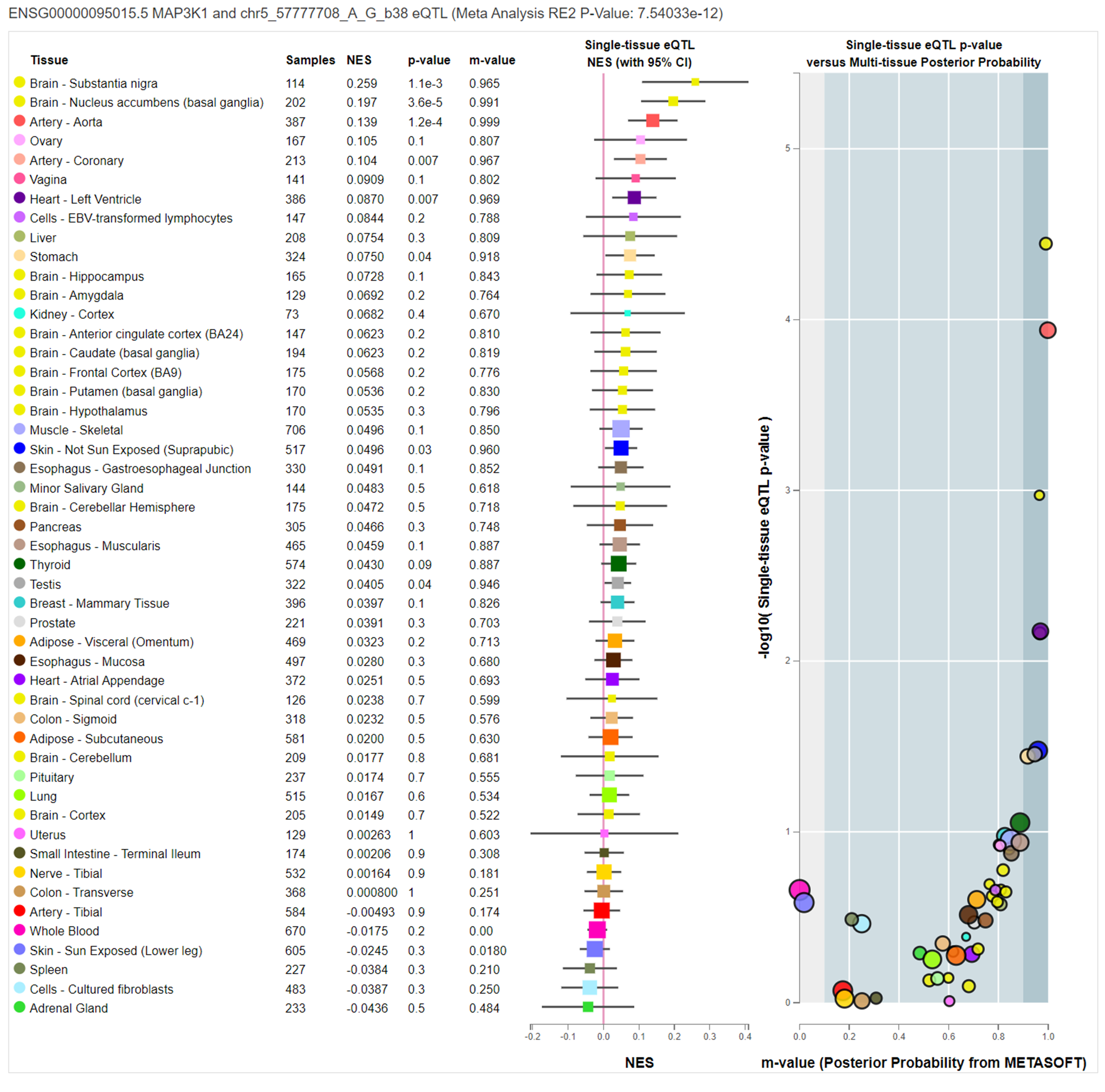

Supplement: Supplementary file 9 — Supplementary Figure 2 [file 41398_2022_2074_MOESM9_ESM.tif]

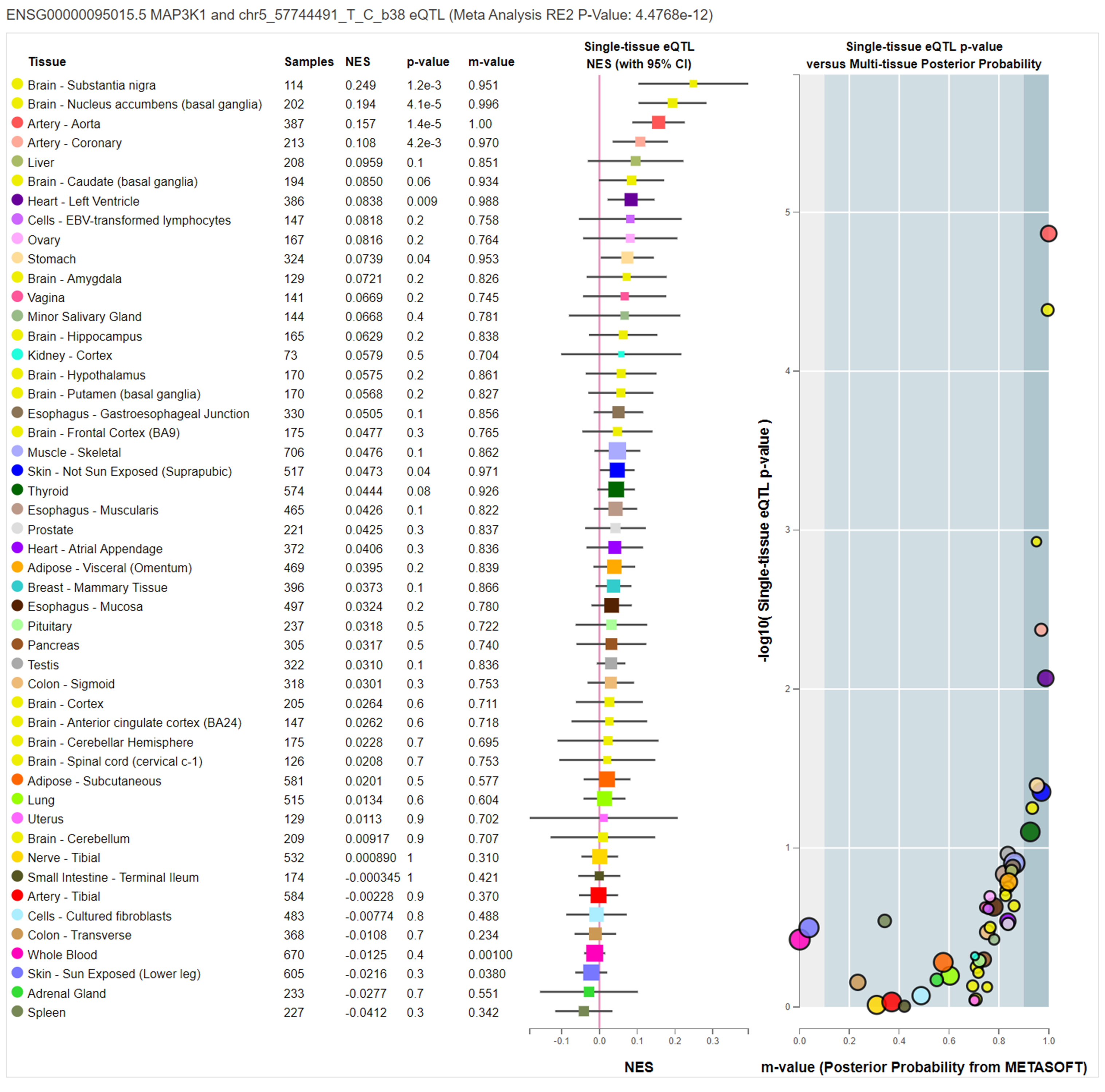

Supplement: Supplementary file 10 — Supplementary Figure 3 [file 41398_2022_2074_MOESM10_ESM.tif]

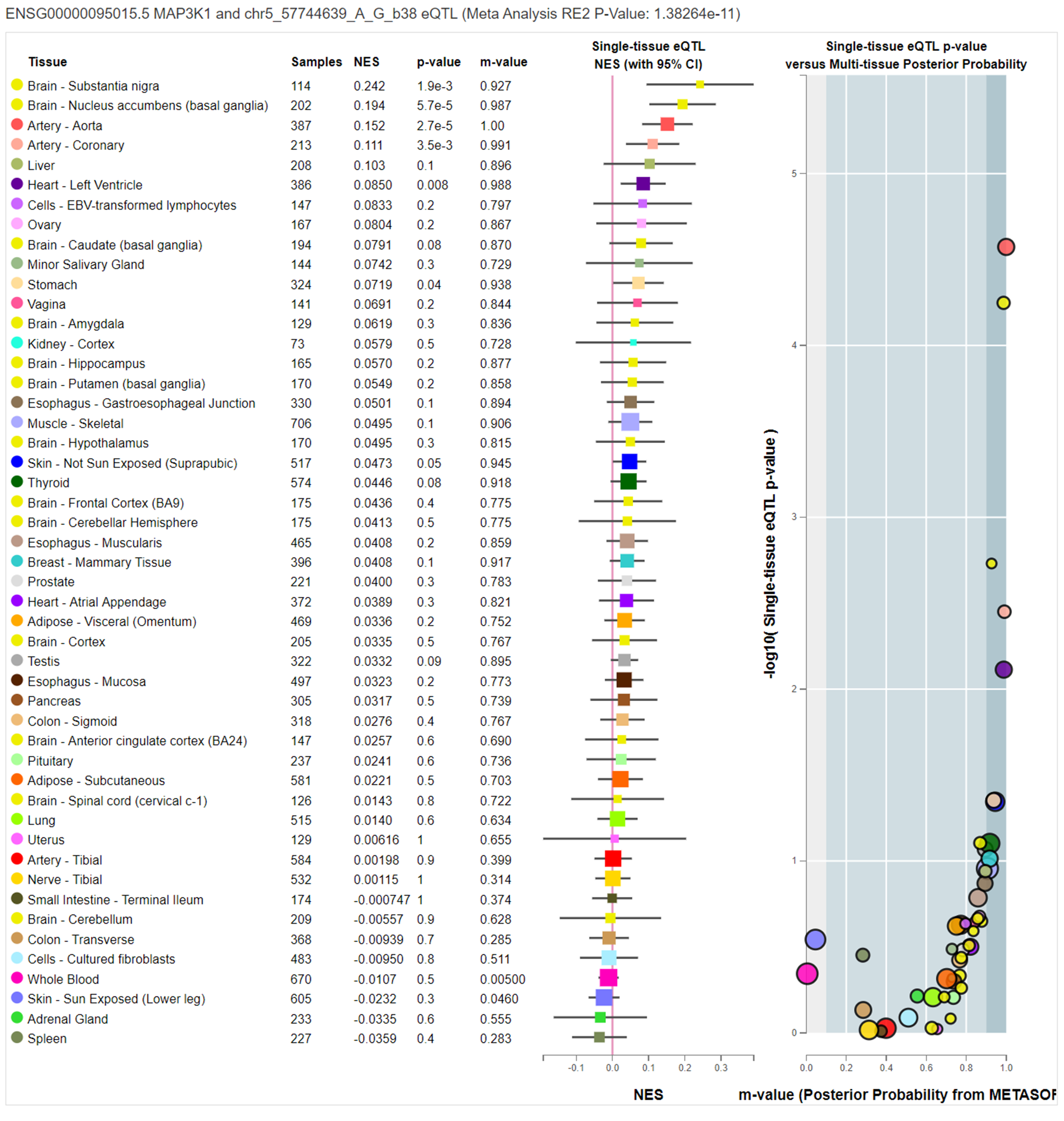

Supplement: Supplementary file 11 — Supplementary Figure 4 [file 41398_2022_2074_MOESM11_ESM.tif]

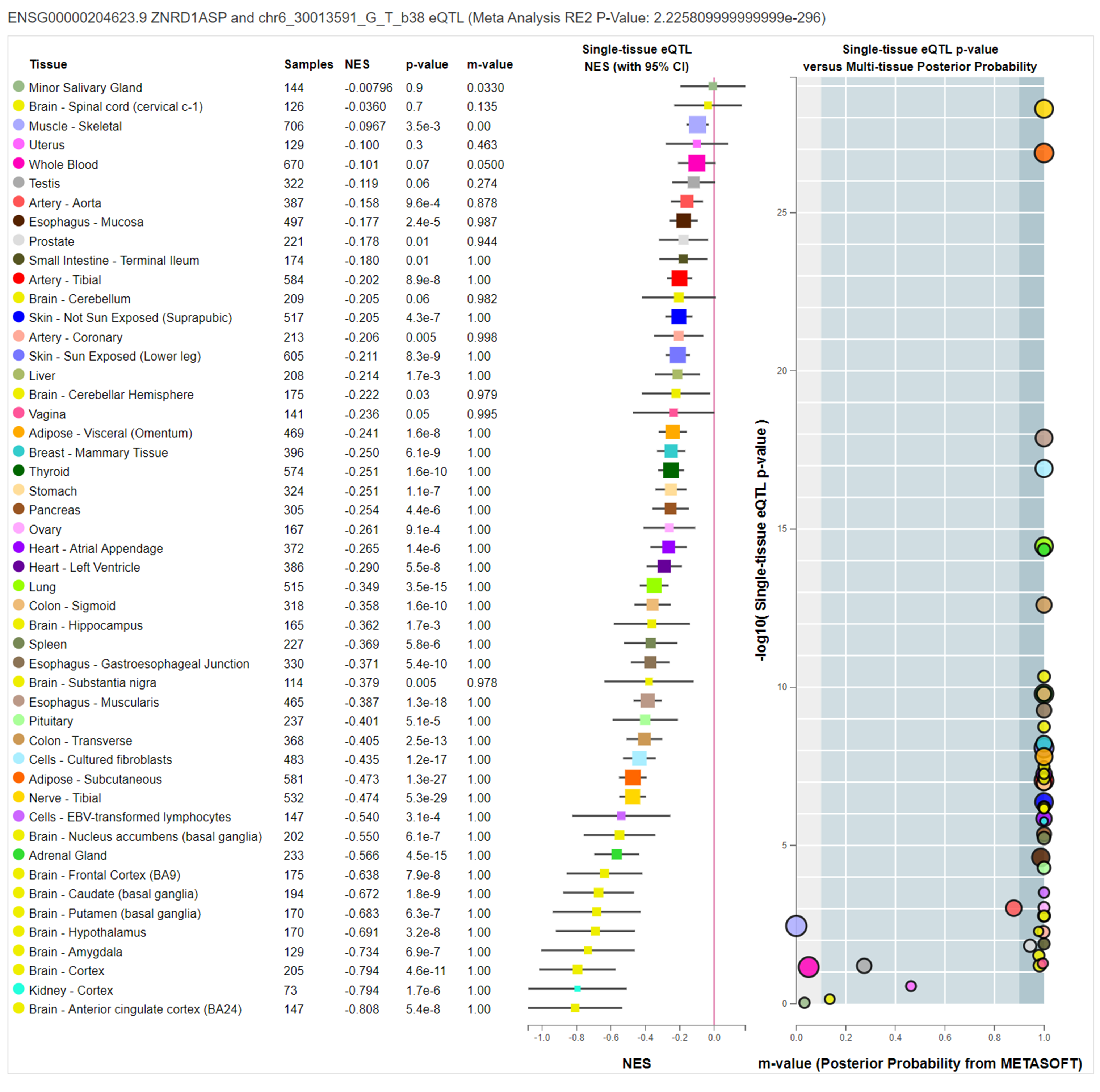

Supplement: Supplementary file 12 — Supplementary Figure 5 [file 41398_2022_2074_MOESM12_ESM.tif]
